# HIF-1α is involved in blood–brain barrier dysfunction and paracellular migration of bacteria in pneumococcal meningitis

**DOI:** 10.1007/s00401-020-02174-2

**Published:** 2020-06-11

**Authors:** Gayatri Devraj, Sylvaine Guérit, Jana Seele, Daniel Spitzer, Jadranka Macas, Maryam I. Khel, Roxana Heidemann, Anne K. Braczynski, Wibke Ballhorn, Stefan Günther, Omolara O. Ogunshola, Michel Mittelbronn, Uwe Ködel, Camelia M. Monoranu, Karl H. Plate, Sven Hammerschmidt, Roland Nau, Kavi Devraj, Volkhard A. J. Kempf

**Affiliations:** 1 Institute for Medical Microbiology and Infection Control, Goethe University, Frankfurt am Main, Germany; 2 Edinger Institute/Neurological Institute, Goethe University, Frankfurt am Main, Germany; 3 Institute of Neuropathology, University Medical Center, Göttingen, Germany; 4 Department of Geriatrics, Evangelisches Krankenhaus, Göttingen-Weende, Germany; 5 Department of Neurology, Goethe University, Frankfurt am Main, Germany; 6 Department of Neurology, Technische Hochschule University Hospital, Aachen, Germany; 7 Max Planck Institute for Heart and Lung Research, Bad Nauheim, Germany; 8 Institute of Veterinary Physiology, University of Zurich, Zurich, Switzerland; 9 Luxembourg Centre of Neuropathology (LCNP), Luxembourg, Luxembourg; 10 Laboratoire National de Santé (LNS), Dudelange, Luxembourg; 11 Luxembourg Centre for Systems Biomedicine (LCSB), University of Luxembourg, Luxembourg, Luxembourg; 12 NORLUX Neuro-Oncology Laboratory, Luxembourg Institute of Health (LIH), Luxembourg, Luxembourg; 13 Department of Neurology, Ludwig-Maximilians University, Munich, Germany; 14 Department of Neuropathology, Institute of Pathology, Julius Maximilians University, Würzburg, Germany; 15 Frankfurt Cancer Institute (FCI), Goethe University, Frankfurt am Main, Germany; 16 Department of Molecular Genetics and Infection Biology, Interfaculty Institute for Genetics and Functional Genomics, Center for Functional Genomics of Microbes, University of Greifswald, Greifswald, Germany

**Keywords:** HIF-1α, VEGF, Dexamethasone, Meningitis, Permeability, *S. pneumoniae*, Blood–brain barrier (BBB), Endothelium, Paracellular transmigration

## Abstract

Bacterial meningitis is a deadly disease most commonly caused by *Streptococcus pneumoniae*, leading to severe neurological sequelae including cerebral edema, seizures, stroke, and mortality when untreated. Meningitis is initiated by the transfer of *S. pneumoniae* from blood to the brain across the blood–cerebrospinal fluid barrier or the blood–brain barrier (BBB). The underlying mechanisms are still poorly understood. Current treatment strategies include adjuvant dexamethasone for inflammation and cerebral edema, followed by antibiotics. The success of dexamethasone is however inconclusive, necessitating new therapies for controlling edema, the primary reason for neurological complications. Since we have previously shown a general activation of hypoxia inducible factor (HIF-1α) in bacterial infections, we hypothesized that HIF-1α, via induction of vascular endothelial growth factor (VEGF) is involved in transmigration of pathogens across the BBB. In human, murine meningitis brain samples, HIF-1α activation was observed by immunohistochemistry. *S. pneumoniae* infection in brain endothelial cells (EC) resulted in in vitro upregulation of HIF-1α/VEGF (Western blotting/qRT-PCR) associated with increased paracellular permeability (fluorometry, impedance measurements). This was supported by bacterial localization at cell–cell junctions in vitro and in vivo in brain ECs from mouse and humans (confocal, super-resolution, electron microscopy, live-cell imaging). Hematogenously infected mice showed increased permeability, *S. pneumoniae* deposition in the brain, along with upregulation of genes in the HIF-1α/VEGF pathway (RNA sequencing of brain microvessels). Inhibition of HIF-1α with echinomycin, *si*RNA in bEnd5 cells or using primary brain ECs from HIF-1α knock-out mice revealed reduced endothelial permeability and transmigration of *S. pneumoniae*. Therapeutic rescue using the HIF-1α inhibitor echinomycin resulted in increased survival and improvement of BBB function in *S. pneumoniae*-infected mice. We thus demonstrate paracellular migration of bacteria across BBB and a critical role for HIF-1α/VEGF therein and hence propose targeting this pathway to prevent BBB dysfunction and ensuing brain damage in infections.

**Electronic supplementary material:**

The online version of this article (10.1007/s00401-020-02174-2) contains supplementary material, which is available to authorized users.

## Introduction

Meningitis is a life-threatening infection of the central nervous system (CNS). Despite advances in antimicrobial therapy, bacterial meningitis remains a serious threat to global health [54]. The most frequently detected bacterial species causing meningitis is *S. pneumoniae*, generally colonizing the upper respiratory tract [52, 54]. Invasive pneumococcal infections present mainly in young children, the elderly and immunocompromised individuals. To cause bacterial meningitis, *S. pneumoniae* must first colonize the nasopharynx to gain access to the intravascular space by breaching the mucosal epithelial layer. Survival in the blood stream, translocation of the bacteria across of the blood–cerebrospinal fluid barrier (BCSFB) or the blood–brain barrier (BBB) and replication within the CNS ultimately cause meningitis that can lead to severe cerebral edema, increased intracranial pressure, seizures, and stroke [49]. The BBB protects and maintains homeostasis in the CNS and is formed by brain microvascular endothelial cells (ECs) whose function is regulated by pericytes, astrocytes, and microglia that together with neurons form the neurovascular unit (NVU) [46]. Vascular damage has been reported as the key pathogenic process, leading to pneumococcal meningitis [22]. However, there is only a slight information on the pathogenic mechanism *S. pneumoniae* exploit to breach the BBB to cause meningitis [24]. Current treatment strategies include administration of high-dose antibiotics to control infection and adjuvant corticosteroids to reduce inflammation and alleviate BBB dysfunction and thereby to reduce edema. In many cases, controlling cerebral edema and intracranial pressure is the prime therapeutic goal. The beneficial effects of adjunctive corticosteroid therapy, primarily dexamethasone, are however inconclusive [4, 71, 22, 63]. Therefore, it is crucial to understand the molecular mechanisms leading to transmigration of *S. pneumoniae* across the BBB into the CNS to identify novel therapeutic targets for bacterial meningitis.

*S. pneumoniae* shows a tropism for endothelial cells mediated by several pathogenicity factors. The pneumococcal adherence and virulence factor A (PavA) have been shown to modulate adherence to host tissue, including brain ECs [5, 56], whereas neuraminidase A (NanA), a surface-anchored sialidase, has been shown to contribute to adherence to human brain microvascular ECs [69]. More recently, the essentiality of teichoic acids for EC adherence and virulence of *S. pneumoniae* has been reported [31]. In addition, pneumococcal adhesins (RrgA and PspC) have been shown to interact with the polymeric Ig receptor and PECAM at the BBB [34]. While these studies demonstrate the mechanism of bacterial adherence to the endothelium, the molecular pathways of the host endothelium involved in invasion of bacteria across the endothelial barrier and the route of transfer, i.e., paracellular versus transcellular, are still poorly understood [18, 60]. We have previously reported that HIF-1α activation is a general phenomenon in infections with subsequent VEGF secretion [14, 36, 75]. VEGF itself—also known as “vascular permeability factor” (VPF)—is responsible for breakdown of BBB function in, e.g., brain tumors and ischemic injury [23, 44, 50, 51]. Furthermore, elevated VEGF levels were shown in meningitis cerebrospinal fluid (CSF) samples [72]. We therefore hypothesized a critical role of the HIF-1α/VEGF signaling in the migration of *S. pneumoniae* across the BBB subsequently causing meningitis.

To investigate the role of HIF-1α/VEGF pathway in migration of *S. pneumoniae* across the BBB, we analyzed mouse and human meningitis specimen for HIF-1α activation. Infection of brain ECs with *S. pneumoniae* followed by HIF-1α/VEGF expression and EC permeability was assessed in vitro. To elaborate the route of bacterial translocation across the endothelium, localization of *S. pneumoniae* was assessed by confocal, super-resolution and live-cell imaging in brain ECs. To analyze the mechanisms of *S. pneumoniae* transfer in vivo, permeability analysis and bacterial presence were assessed, followed by electron microscopy of hematogenously infected mice. Isolated brain microvessels from infected mice were subjected to RNA sequencing to assess regulation of the HIF-1α/VEGF pathway. The contribution of HIF-1α on *S. pneumoniae*-induced permeability was analyzed in loss-of-function (LOF) experiments using HIF-1α inhibitor echinomycin and in brain ECs from HIF-1α KO mice. Finally, therapeutic rescue experiments were performed using echinomycin in *S. pneumoniae*-infected mice for survival analysis and analysis of BBB function by immunohistochemistry.

## Materials and methods

### Animal care and handling

Adult (3–6 months old) wild-type C57BL/6 mice of both sexes were used in the study unless otherwise specified. Transgenic HIF-1α^flox/flox^ mice of the same age group were also used post-PCR validation of the genotype [62]. The number of animals and number of experiments are indicated where appropriate. All animals were sacrificed under anesthesia and their number was kept to a minimum based on extracted tissue/cell amount and based on statistically appropriate sample size. Mice were handled according to the German Protection of Animals Act and in compliance with ARRIVE (animal research: reporting of in vivo experiments) guidelines and principles of laboratory animal care (NIH publication No. 86-23, revised 1985). The local committee regulations were strictly followed for the approved animal experiments (Approval # 33.9.42502-04-14/1553 by the Animal Ethics Committee of Lower Saxony State Office for Consumer Protection and Food Safety (LAVES) to R. Nau and approval # 55.2-1-54-2532-78-2012 by the Committee on the Ethics of Animal Experiments of the Government of Upper Bavaria to U. Koedel).

### Human meningitis specimen

All the studies on human subjects were approved by an ethics statement (GS-249/11 for autopsy material and GS-04/09 for resection material, to K. Plate, Goethe University Hospital, Frankfurt, Germany). Tissue was formalin fixed, paraffin embedded and utilized in immunohistochemistry analysis. Relevant anonymized patient data are included in Table 1.

**Table 1:** Quantification and patient data of human meningitis cases

Case Id	Age	Gender	Meningitis pathogen	Number of HIF-1α + cells		Signal
				Cortex	Leptomeninges	
1	64	Male	*S. pneumoniae*	0	0	
2	61	Male	*S. pneumoniae*	35	650	+++
3	88	NA	*S. pneumoniae*	23	30	+
4	63	Male	*S. pneumoniae*	150	88	++
5	24	Male	*S. pneumoniae*	NC	NC	++
6	71	Male	*M. tuberculosis*	NC	NC	++ to +++
7	67	Female	*Fungi*	NC	NC	++ to +++
8	72	Male	*T. gondii*	NC	NC	++ to +++
9	28	Female	*C. neoformans*	NC	NC	++ to +++

Ten areas in the cortex and five in the leptomeninges were counted for HIF-1α-positive cells from each specimen in five pneumococcal meningitis cases. The patient data for these cases and other cases of meningitis examined (supplementary Fig. 2, online resource) are included. NA indicates data not available and NC indicates not counted. For some cases, the staining signal was weak (indicated by +). Cause of death for all the patients from (1–8) was meningitis, but for patient 9 it is not available

### Immunohistochemistry from mouse/human meningitis samples

Formalin-fixed paraffin-embedded mouse meningitis samples and autopsy samples from human meningitis cases (including bacterial, viral, fungal or parasitic meningitis infections) were collected to investigate HIF-1α activation by immunohistochemistry. In brief, paraffin-embedded samples were cut into 3–5 µm thin sections on a Leica microtome (SM2000R, Leica Microsystems, Germany) and were deparaffinized and rehydrated in decreasing ethanol concentration prior to different stainings. Hematoxylin and eosin (H & E) stainings were used for the histopathological examination and diagnostics. Single or double stainings using various antibodies (Table 2) were performed by standard protocols on a Ventana Discovery XT automated immunohistochemistry system (Roche, Switzerland).

**Table 2:** Antibodies used for Western blotting (WB) and immunohistochemistry (IHC)

Antibody	Application	Company	Reference	Dilution
α-Tubulin (mouse)	WB	Sigma-Aldrich	T6199	1:500
β-Actin (mouse)	WB	Santa Cruz	sc-69879	1:500
VE-cadherin (goat)	WB/IHC	Santa Cruz	sc-6458	1:200
Claudin-5 (mouse)	WB/IHC	Invitrogen	352500	1:200
CD31 (rat)	IHC	Dianova	DIA310	1:50
Occludin (mouse)	IHC	Invitrogen	331500	1:200
ZO-1 (rabbit)	IHC	Invitrogen	617300	1:200
Podocalyxin (goat)	IHC	R&D Systems	AF1556	1:200
HIF-1α (rabbit)	WB/IHC	Novus Biologicals	NB100-134	1:200
Pneumococcal (rabbit)	IHC	Dr. Hammerschmidt	NA	1:500
Donkey anti-goat 555	IHC	ThermoFisher	SA5-10087	1:500
Donkey anti-rabbit 488	IHC	ThermoFisher	SA5-10038	1:500
Donkey anti-mouse 555	IHC	ThermoFisher	SA5-10167	1:500
Donkey anti-mouse 680	WB	LI-COR	925-68072	1:2000
Donkey anti-rabbit 800	WB	LI-COR	925-32213	1:2000
Donkey anti-goat 800	WB	LI-COR	925-32214	1:2000

### Bacterial strains and culture conditions

*S. pneumoniae* serotype 2 strains D39 (NCTC 7466), D39Δ*cps*, HlpA-GFP-cat modified D39 strain ([40], and serotype 4 strains TIGR4 and TIGR4Δ*cps* were used as described previously [58, 75]. Frozen vials of *S. pneumoniae* were thawed and cultured on Columbia blood agar plates (Oxoid, 5% sheep blood) for 8–10 h at 37 °C and passaged for 12–14 h. *S. pneumoniae* from blood agar plate were resuspended in Todd–Hewitt broth (Oxoid, UK) supplemented with 0.5% yeast extract (THY) to mid-log phase for use in infection experiments. Growth rates were quantified by measuring optical density (NanoPhotometer® P-Class, Implen, Germany) and by counting colony forming units (CFU) [56].

### Culture of bEnd5 cells

Immortalized mouse brain endothelioma cells (bEnd5 [61]) were grown in DMEM (GlutaMAX™, high glucose, pyruvate containing media, Gibco, Germany) supplemented with 10% FBS (Sigma, Germany), β-mercaptoethanol (75 µM), 1% nonessential amino acids, 100 U/ml penicillin and 100 µg/ml streptomycin, in a humidified incubator at 37 ºC with 5% CO_2_. T75 flasks were coated with gelatin solution (0.1%) for 1 h at 37 ºC and bEnd5 cells were plated at a density of 25,000/cm^2^ and passaged after 7–8 days by 0.05% trypsin/EDTA treatment with media change every 2–3 days.

### Isolation and culture of primary mouse/human brain microvascular ECs

Primary mouse brain microvascular endothelial cells (MBMECs) were isolated from adult WT C57BL/6 or HIF-1α^flox/flox^ mice and cultured exactly as described previously [27]. Cultures were maintained in MCDB-131 complete medium (MCDB-131 (Gibco, Germany), 2 mM l-glutamine (Gibco), 20% FBS (Biochrom), 0.01% heparin (Sigma), 2 mM penicillin–streptomycin solution (Gibco), 0.1% NaHCO_3_ and 0.05 mg/ml endothelial cell-growth supplement ECGS (lyophilized powder prepared from homogenized porcine brains obtained from slaughterhouse [2]). Preparations were quality controlled by spindle morphology of ECs and by immunocytochemistry for EC markers (claudin-5, VE-cadherin) [13]. Primary human brain microvascular endothelial cells (HBMECs) were obtained at passage 2 (Pelobiotech, Germany) and cultured as described above for MBMECs and used within passage #5.

### Culture of primary human pericytes and primary mouse astroctyes

Primary human pericytes (passage #5–8, ScienCell, USA) and primary mouse astrocytes (isolated from pups as described previously [27, 70], passage #1–2) were cultured in their respective media (pericyte medium, ScienCell) or astrocyte medium comprising DMEM (high glucose, GlutaMAX™, pyruvate, Gibco®), 10% FBS, 100 U/ml penicillin and 100 µg/ml streptomycin.

### Detection of cellular hypoxia in vitro

Cellular hypoxia was investigated in bEnd5 cells upon *S. pneumoniae* infection using the hypoxia-sensitive marker pimonidazole hydrochloride (Hypoxyprobe™ Red549 kit, Hypoxyprobe, USA). Cellular hypoxia was detected by adding 200 mM pimonidazole hydrochloride to bEnd5 cells 30 min prior to infection. Pimonidazole–protein adducts formed (proportional to level of hypoxia) were detected by immunofluorescence staining according to the manufacturer’s protocol.

### Oxygen measurement assay in vitro

The O_2_ concentration in the culture medium of infected bEnd5 cells was measured using the SensorDish® reader (SDR, Germany). Cells were directly seeded on the OxoDish® (OD) 24-well polystyrene dish. Upon infection, the dish was placed over the SDR and equilibrated to CO_2_ (5%) and O_2_ (19%) levels set within a tissue-culture incubator at 37 °C. Pre-calibrated oxygen sensors integrated at the bottom of each well allow for non-invasive contactless read-out in the SDR through the transparent OD plate bottom. Oxygen level was equilibrated for 1 min at 37 °C, followed by measurement every 5 min up to 3 h post-infection and O_2_ concentration in the medium was obtained (pO_2_% air saturation).

### Western blotting

Culture (6–7 days) and infection of bEnd5 cells were performed in six-well plates as described above. At the end point, cells were washed in ice-cold PBS, followed by harvesting them in HEPES EDTA sucrose buffer (HES, 10:1:250 mM, pH 7.4) containing protease and phosphatase inhibitors. Western blotting was performed as described previously with a few modifications [12, 13]. Cell suspensions in HES were sonicated and aliquoted for western blotting and protein assay and stored at − 80 °C until use. Protein concentration was measured by BCA assay (Thermo Fisher) and equal amounts of proteins were solubilized (2.3 M urea, 1.5% SDS, 50 mM Tris, 25 mM TCEP and 0.01% BPB) for 1 h at 30 °C on a shaker and centrifuged at 15000×*g* for 5 min at RT to remove insoluble debris. Equal volumes of solubilized supernatants were loaded onto 8–12% Tris–HCL bis-acrylamide gels and subjected to electrophoresis (80 V, 3 h, RT). The gels were subsequently transferred overnight in Tris–glycine buffer containing 20% methanol without SDS (8 °C, 36 V). The membranes were subjected to Ponceau staining to confirm transfer and equal loading of proteins. The membranes were then blocked (1 h, RT), followed by primary antibody incubation overnight (8 °C) in 1 × Roti®-block buffer (Roth). The washed membranes (3 × 5 min in 0.1% Tween/PBS) were subjected to secondary antibody incubation in the same buffer, rinsed in PBS and imaged by Odyssey Fc imager (Li-COR). The acquired images were analyzed with Image Studio 2.1 (Li-COR). Quantification of pixel intensity was performed from equal-area rectangles drawn around bands of interest (subtracting background from an empty lane), normalizing to the values of β-actin (housekeeping protein) and presented as percentage of control. Antibodies used and their dilutions are included in the Table 2.

### Quantitative RT-PCRs

Samples taken from the in vitro permeability assays were used for qRT-PCR for direct comparison to functional data. Total RNA was extracted using RNeasy micro or mini kit including RNase-free DNase set (Qiagen, Germany) for inhibiting residual DNase activity. The cDNA was generated using RevertAid H-Minus First Strand cDNA synthesis kit (ThermoScientific, Germany) according to the manufacturer’s protocol. Quantitative RT-PCR was performed using ABsolute qPCR SYBR Green Fluorescein Mix (Thermo Scientific, Germany) in a LightCycler® 480 Real-Time PCR Detection System (Roche, Germany) using the following conditions: 15 min at 95 ºC, 45 cycles of (30 s at 95 ºC, 30 s at 61 ºC, and 35 s at 72 ºC), 1 min at 95 ºC, 1 min 61 ºC, and for melt curves 80 cycles at a set point of 55 ºC with 0.5 ºC step and 94.5 ºC exit temperature. LightCycler® 480 instrument software version 1.5 (Roche, Switzerland) was used for analyzing the RT-PCR data and exported into Excel (Microsoft Office, USA) for quantification of expression values (R) according to 2^−∆∆Ct^ method using RPLP0 and G6PDX as housekeeping genes. Primers were designed using primer3 software (https://bioinfo.ut.ee/primer3-0.4.0/) from mouse or human mRNA refseq ID from NCBI and validated in efficiency curves before use. Primers sequences are included in Table 3.

**Table 3:** Mouse-specific primers used for qRT-PCR analysis

Gene ID	Sequence 5′–3′ sense	Sequence 5′–3′ e antisense
cdh5	gcccagccctacgaacctaaa	gggtgaagttgctgtcctcgt
hif1a	catccatgtgaccatgaggaaa	aatatggcccgtgcagtgaag
vegf	ccgcagacgtgtaaatgttcct	ttccggtgagaggtctggttc
rplpo	gtgtttgacaacggcagcatt	tctccacagacaatgccagga

### Dextran-based transwell permeability assay in BBB infection models

Cells (bEnd5, MBMEC or HBMEC) were grown in transwell chambers (Fig. 3a) as described earlier with slight modifications [8, 27]. Approximately 100,000 cells/cm^2^ were seeded on fibronectin- (5 µg/cm^2^, Sigma Aldrich, Germany) coated 24-well plate cell-culture inserts (PET 1.0 micron, Greiner Bio-one, Germany) and cultured in antibiotic-free complete medium (described above for the particular cell type) for 3–4 days for primary brain ECs and 5–6 days for bEnd5. Bacteria were thawed (*S. pneumoniae* D39 and TIGR4 strains) and centrifuged at 2500×*g* for 5 min at room temperature, washed with PBS and re-suspended in the infection medium (antibiotic-free complete medium containing 1% FBS). Dextrans (3 kD tetramethylrhodamine (TMR)/70 kD fluorescein isothiocyanate (FITC, ThermoFisher) were added to the infection medium (10 µM) and cells infected apically for 3–6 h at multiplicity of infection (MOI) of 50 in a total volume of 200 µl, whereas the basal chamber contained 600 µl of the infection medium. Media aliquots (100 µl) were collected at designated time points and twofold 100% ethanol was added to kill bacteria and 200 µl was transferred to black-well plates (Greiner Bio-One, Germany) for dextran permeability assessment. The following excitation/emission (nm) was utilized for reading the fluorescence: TMR 3 kD dextran (550/580), followed by FITC 70 kD dextran (490/520) in Infinite M-200 PRO fluorescence plate reader (Tecan). For permeability assessment, the raw fluorescence units (RFU) obtained for the bottom chamber were normalized to the top chamber and expressed as percentage relative to control condition as described previously [8].

### Dextran-based transwell permeability assays using conditioned media

Primary human pericytes and primary mouse astrocytes were cultured until confluency and infected with *S. pneumoniae* (MOI 50, 3 h). The media supernatant along with bacteria was collected, filtered (0.2 µm) and stored at − 80 °C. The influence of pneumococcal infections of astrocytes and pericytes on the endothelial barrier was assessed by treatment of bEnd5 cells plated on inserts with the conditioned media (diluted 1:3 with fresh medium). Permeability assays were performed as described above.

### Transendothelial electric resistance (TEER) measurements

Transwell inserts (1 µm-pore 24-well PET) were coated with fibronectin and bEnd5 cells (100,000/cm^2^) were seeded and transferred to a cellZscope® device (NanoAnalytics, USA) placed in a humidified incubator (37 °C, 5% CO_2_). TEER values were obtained from continuous impedance measurements as described previously [8]. Once a plateau was reached in TEER levels (typically after 4–5 days), cells were infected with D39 (MOI: 100) in the infection medium as described above and TEER measurements were obtained for 8 h post-infection. TEER data were obtained in ohm cm^2^, followed by setting the value of the control group between 3–4 h post-infection to 100% within the cellZscope software (v2.2) for ease of analysis. These data and representative images were exported for subsequent analysis.

### Bacterial transmigration assays

Infection of bEnd5 cells was performed exactly as described above for permeability assays (Fig. 3a, b), but using 3.0 µm pore inserts to allow bacterial transmigration. Media collected from the bottom chamber of duplicate wells (1.2 ml) were pooled, centrifuged (3000×*g*, 10 min, RT) and resuspended in 100 µl of DMEM infection media without antibiotics. Serial dilutions (10^0^–10^5^) of the bacterial suspension were plated in triplicate on blood agar plates and incubated at 37 °C overnight. Aliquots from inserts were also plated as a control for the assay. Quantification of microorganisms was performed by visually counting the number of CFU in each dish and expressed as log_10_ CFU/ml.

### Immunofluorescence staining of cultured brain ECs

Primary mouse brain ECs (MBMEC) or bEnd5 cells on inserts were washed twice with cold 1×PBS and fixed with − 20 °C cold methanol for 3 min at RT, followed by a PBS wash. Fixed cells on inserts were then blocked and permeabilized for 45 min at RT in PBS containing 1% BSA and 0.5% Triton X-100, pH 7.5. Primary antibody staining was performed for 1 h at RT in the antibody incubation buffer (PBS containing 0.5% BSA, 0.25% Triton X-100, pH 7.2), followed by 3 × 5 min washes in PBS. Species-specific fluorescent secondary antibodies were used in combination with DAPI (0.3 µM) and incubated for 1 h at RT, followed by washes in PBS (3 × 5 min). Inserts were then cut out and mounted on glass slides using Fluroprep (bioMérieux, Germany) or Aqua-Polymount (Polysciences Inc., Germany). Slides were kept for drying overnight at RT in dark, followed by visualization and image acquisition using a wide-field microscope (Nikon 80i) or a confocal fluorescence microscope (Nikon C1si). The same procedure was also performed for MBMECs or HBMECs that were cultured in fibronectin-(5 µg/cm^2^) coated chamber slides (8-well, cover glass II, Sarstedt) suitable for super-resolution microscopy (SRM) and imaged directly in PBS after staining using Nikon N-SIM microscope (Nikon Apo TIRF 100× objective, Germany) for SRM or Nikon C1si for confocal imaging. Images were processed using NIS Elements imaging software (version 4, Nikon) and saved in the raw ND2 format and exported as TIFF files. Antibodies and their dilutions are included in Table 2.

### Immunofluorescence staining and quantification for paraffin-embedded brain tissues

PFA (4%) fixed paraffin-embedded brain sections (3–5 µm) were also subjected to immunofluorescence staining after standard deparaffinization protocol. Epitope retrieval was performed for 45 min in boiling citrate buffer (1 × AR6, PerkinElmer). After cooling down for 30 min at RT, slides were washed with PBS, followed by blocking/permeabilization in PBS containing 0.5% BSA and 0.2% Triton X-100, pH 7.4. Sections were encircled with a PAP pen to create a hydrophobic barrier, followed by adding 70–80 µl of the primary antibody mix and slides incubated O/N at 4 °C in a humidified chamber. After three washes, 5 min each in PBS/0.2% Triton, species-specific fluorescent secondary antibody mix containing DAPI (1:500) was added and the slides incubated for 1 h at RT. After two washes in the above wash buffer, two final washes were performed in PBS followed by mounting the slides with Aqua Polymount (Polysciences Inc.) and allowed to dry at least O/N in dark at RT before proceeding to microscopy. Antibodies and their dilutions are included in Table 2. Images were acquired using a wide-field microscope (Nikon 80i) using 60× water immersion objective with set exposure and gain settings across samples for a particular antibody combination. For the purpose of quantification of expression intensity, four images were obtained for each animal for a particular antibody staining, utilizing two sections and acquiring two images/section from frontal and occipital cortical regions. Using the NIS-Elements software (version 5, Nikon), the acquired raw ND2 image files were subjected to binary thresholding using whole image as the region of interest (ROI). Measurement was then performed for binary area, mean fluorescence intensity, and number of objects applying 1 × clean function, 4 × separation as well as size range of 3–9 µm for objects. Colocalization analysis was performed from overlapping binary area between corresponding channels using intersection function in the software. All binary layers including intersection layers were stored within the original ND2 files. Values from each of the four images for an animal per antibody combination for all channels including co-localization were exported to a spreadsheet (MS Office Excel). Immunohistochemistry (IHC) expression intensity was obtained in arbitrary units (a.u) as the product of thresholded binary area and the mean fluorescence intensity. IHC cell number was obtained as the number of objects in the thresholded image. Co-localization IHC intensity was obtained as the product of overlapping binary area between channels with the mean intensity of channel of interest. The same intersection analysis also yielded the number of co-localized objects. Data were organized in the spreadsheet such that a row represented animal ID and replicates from the four images were in four adjacent columns. This data was then imported to Prism software (v5, GraphPad) into a grouped data table with four sub-replicate columns followed by graphing and statistical analysis comparing two groups at a time. For representative images, confocal laser scanning microscopy (Nikon C1si) was performed using either 60× or 100× objective (zoom factor 2 for scanning) and images exported as high-resolution TIFF files.

### Live-cell imaging of *S. pneumoniae*-infected brain ECs

Primary brain microvascular ECs (MBMEC) were isolated from three adult wild-type mice and plated directly on an eight-well chamber slide with glass bottom (Ibidi) precoated with rat-tail collagen (Corning) as described for MBMEC isolation. ECs from each animal were plated onto two wells with a growth area of 1 sq cm per well. After 4 h, the medium was changed to puromycin containing MCDB-131 complete medium as described in the MBMEC isolation and cultured for 2 days. On day 3, cells were infected with GFP-labeled D39 *S. pneumoniae* strain [40] at an MOI of 10 using 20 µl of 5 × 10^6^ CFU/ml bacteria in 0.3 ml media per well. Live-cell imaging was initiated 1 h post-infection on a Nikon Eclipse Ti microscope fitted with an incubation chamber attached to temperature and CO_2_ controllers (Okolab). The temperature was maintained at 37 °C by a heated water bath (E200, Lauda) attached to its controller. The CO_2_ level was maintained at 5% by its controller attached to a bubbler installed in the water bath mixed with CO_2_ from an external tank utilizing an air pump (Okolab). Images were acquired using DS-Qi2 camera (Nikon) at 10 s intervals simultaneously in bright field and GFP fluorescence channels for a duration of 2 h using 40× Plan Apo objective, setting an additional 1.5× zoom on the microscope for all lenses to obtain a total 60× magnification. The saved raw ND2 files were processed in NIS-Elements software (version 5, Nikon) for regions/time intervals of interest and exported as TIFF image files and MP4 format video files.

### Murine infection models and downstream processing

Three mouse pneumococcal infection models—infection via intracerebral, intracisternal and via intraperitoneal routes—were utilized in this study. For intracerebral infection, mice received 10 µl of 10^5^ CFU/ml of *S. pneumoniae* D39 after i.p anesthesia with ketamine (100 mg/kg) and xylazine (10 mg/kg) as described previously [32]. For intracisternal infection, 15 µl of 10^7^ CFU/ml of *S. pneumoniae* D39 strain was injected under brief anesthesia with isofluorane [6]. Animals were sacrificed between 24 and 48 h post-infection and brains were extracted and formalin fixed, followed by embedding in paraffin for use in immunohistochemistry. Hematogenous infection was induced by intraperitoneal injection of 100 µl of 10^7^ CFU/ml *S. pneumoniae* D39 strain [68, 74]. Animals were anesthetized 18 h post-infection, followed by transcardial perfusion with PBS as described previously [15] and used in permeability assay, EM analysis or for brain microvessels isolation [27].

### In vivo tracer permeability assay

Permeability assays were performed as described previously [15] in infected or healthy animals (Fig. 4a). TMR 3kD dextran (200 µl of 2 mM stock in PBS, D3308, Thermo Fisher, Germany) was injected intraperitoneally, followed by anesthesia 5 min later. After a circulation time of 20 min for the tracer, the animals were prepared for transcardial perfusion and ~ 300 µl blood was collected from the chest cavity just after atrial puncture, followed by perfusion for 3 min with PBS. Hemibrain (free of the olfactory lobes, cerebellum and hindbrain) and a single kidney were collected and immediately frozen on dry ice and stored at − 80 °C. For fluorescence measurement, samples were thawed on ice, weighed and homogenized in PBS (200 µl for hemi-cerebrum and 300 µl for kidney), followed by centrifugation for 20 min, 4 °C at 15,000×*g*. Supernatants (40 µl) as well as equal volumes of serum (1/5 dilution in PBS) were loaded into a 384-well black plate and fluorescence was measured at the corresponding excitation (550 nm)/emission (580 nm) in a plate reader (Tecan, Switzerland). Sham animals (without tracer injection) were used to subtract autofluorescence values. Permeability index (ml/g) was calculated as the ratio of (tissue RFUs/g tissue weight) to (serum RFUs/ml serum).

### Transmission electron microscopy of brain tissues of *S. pneumoniae*-infected mice

Animals were subjected to intraperitoneal *S. pneumoniae* infection for 18 h, followed by anesthesia and transcardial perfusion with PBS (2 min) and then with 4% PFA/PBS (4 min). The isolated brains were post-fixed with 4% PFA/2% glutaraldehyde overnight at 4 °C. The tissue was cut into small pieces and post-fixed with 1% OsO4 for 2 h at RT, dehydrated in graded acetone and embedded in Durcupan with the polymerization for 72 h at 60 °C. Ultrathin sections were cut (Leica Ultracut UCT, USA), contrast enhanced with lead citrate and uranyl acetate and analyzed using Tecnai Spirit BioTWIN (FEI, ThermoFisher, Germany) electron microscope at 120 kV. Images were acquired with an Eagle 4 K CCD bottom-mount camera (FEI, ThermoFisher, Germany). Artificial coloration was performed using Photoshop CS4 (Adobe).

### Isolation of mouse brain microvessels (MBMVs)

Mice with and without intraperitoneal *S. pneumoniae* infection were anesthetized, followed by transcardial perfusion of PBS as described previously [74]. Brain microvessels were isolated as described previously [12, 13, 27]. Brains were harvested and cerebellum and olfactory lobes were removed, followed by rolling on Whatman filter paper (Schleicher & Schuell, Germany) to peel off meninges. The pooled cerebral hemispheres were homogenized in microvessel buffer (MVB, 15 mM HEPES, 147 mM NaCl, 4 mM KCl, 3 mM CaCl_2_, 1.2 mM MgCl_2_, 5 mM glucose, and 0.5% BSA, pH 7.4) applying 15–20 strokes using a Dounce homogenizer (0.025 mm clearance, Wheaton, USA) attached to an overhead electric stirrer (2000 RPM, 45 W, VOS14, VWR). Samples were then centrifuged at 400×*g* for 10 min at 4 °C and the pellet was resuspended in fourfold 25% BSA in PBS and further centrifuged at 2000×*g* for 30 min to remove myelin fat that was aspirated carefully. The microvessel pellet containing red cells and nuclei was resuspended in MVB and filtered through 100-μm and 40-μm nylon meshes (Becton Dickinson, Germany). Microvessels on top of the 40-μm mesh were washed in MVB and lysed directly on mesh with RLT buffer (Qiagen, Germany) containing 40 mM dithiothreitol (DTT) and stored at − 80 °C until use. The scheme for the isolation is included (Fig. 6a).

### RNA sequencing of brain microvessels from mice infected with *S. pneumoniae*

RNA was isolated from brain microvessels from healthy and intraperitoneally infected mice using the RNAeasy micro Kit (Qiagen, Germany) including a DNase digestion step to eliminate genomic DNA. The quality of RNA and the prepared library was verified on LabChip Gx Touch 24 (Perkin Elmer, Germany). Equal amount of total RNA (200 ng), measured by Qubit 3.0 (ThermoFisher, Germany) was used as input for SMARTer Stranded Total RNA Sample Prep Kit—HI Mammalian (Clontech, USA). Sequencing was performed using v2 chemistry (NextSeq500 instrument (Illumina, USA), resulting in an average of 28 million reads per library (1 × 75 bp single-end setup) that were assessed for quality, adapter content and duplication rates (FastQC: a tool for high throughput sequence data—https://www.bioinformatics.babraham.ac.uk/projects/fastqc). Reaper version 13–100 was employed to trim reads after a quality drop below a mean of Q20 in a window of 10 nucleotides [9], utilizing reads between 30 and 150 nucleotides for further analyses. Trimmed and filtered reads were aligned with the Ensembl mouse genome version mm10 (GRCm38) using STAR 2.4.0a [17] with the parameter “–outFilterMismatchNoverLmax 0.1” to increase the maximum ratio of mismatches to mapped length to 10%. The number of reads aligning to genes was obtained from the Subread package using featureCounts 1.4.5-p1 tool [45], aggregating per gene only those reads mapping at least partially inside exons, whereas those overlapping multiple genes or to multiple regions were excluded. Differentially expressed genes were identified using DESeq2 version 1.62 [48], and genes with a minimum fold change of ± 1.5 (log2 ± 0.59), a maximum Benjamini–Hochberg corrected *p* value of 0.05, and a minimum combined mean of five reads were considered significant for differential expression. Based on the gene identifiers, the Ensemble annotation was enriched with UniProt data (release 06.06.2014). Bioinformatics analyses were performed for enriched pathways and diseases on genes commonly regulated in all four meningitis microvessel samples compared to all the healthy ones (*p* < 0.05, log twofold-change > 0.585). KOBAS web server including KEGG, PANTHER, and Reactome databases [76] was used for these analyses.

### Loss-of-function (LOF) experiments for HIF-1α

Echinomycin (Calbiochem, Germany), a chemical inhibitor of HIF-1α, was used to treat bEnd5 cells at concentration of 100 nM in complete medium 24 h prior to infection, followed by permeability assays. Genetic knockdown experiments were performed in bEnd5 cells using *si*RNA for HIF-1α. Cells were first plated onto inserts as described for transwell permeability assay, followed by transfection with HIF-1α *si*RNA on the next day using Lipofectamine RNAiMax. To 0.5 ml of OptiMEM medium (ThermoFisher, Germany), 10 µl (10 µM stock) each of the two *si*RNAs (Qiagen SI00193011, SI00193018) were added and to another tube containing 0.5 ml OptiMEM 10 µl Lipofectamine RNAiMax (ThermoFisher, Germany) was added. After mixing both tubes individually, they were incubated for 5 min at RT, followed by combining the two tubes and incubation for another 15 min at RT. During the incubation time, cells were washed once with OptiMEM medium and 200 µl of fresh OptiMEM was added to each insert and 1 ml was added to the bottom chamber. After the incubation of the *si*RNA mix, 100 µl was added to each insert and the medium changed to bEnd5 complete medium after 6 h. Infection and permeability/qRT-PCR assays were performed 3 days after transfection as described earlier.

HIF-1α KO in primary mouse brain microvascular ECs was performed as shown in the schematic of Fig. 7d. Briefly, MBMECs were isolated from HIF-1α^flox/flox^ mice. After overnight culture, cells were treated with puromycin (5 µg/ml) for 2 days to obtain pure ECs, as puromycin is toxic to non-ECs. The pure MBMECs from HIF-1α^flox/flox^ mice were then treated with TAT-cre (1 µM, Excellgen, USA) for 2 days in antibiotic-free MCDB-131 complete medium. Cells were then split on to transwell inserts at passage 1 and were used 2 days post-plating for infection and permeability/qRT-PCR assays as described earlier.

### Therapeutic rescue experiments

Adult wild-type female mice were infected with *S. pneumoniae* D39 strain by intraperitoneal (i.p.) injection of 100 µl of 5 × 10^6^ CFU/ml bacteria [68, 74]. Animals were treated with echinomycin (20 µg/kg body weight) by i.p. injection every 12 h starting 4 h post-infection using a 5 µM stock (5%DMSO/95% PBS, 100 µl/20 g mouse). Control animals were injected with 100 µl of just the vehicle (5% DMSO/95% PBS). Clinical scoring was performed in a blinded fashion every 4 h starting 16 h post-infection on a 4-point scale with 1 being the lowest score and 4 highest score indicating severe symptoms as described previously [29]. Animals that had a clinical score of 3 or more indicating severe lethargy and impaired mobility were euthanized and the ones that died in between the scoring time points were given the worst score of 4. This therapeutic survival experiment was conducted for a total duration of 48 h at which time point all surviving animals were sacrificed. Irrespective of the treatment group, at the survival end point, i.e., when the animal died or at the end point of the experiment, i.e., 48 h, the brain was harvested, split at the miline with one hemi-brain stored in 4% PFA on ice and the other hemi-brain embedded natively in O.C.T compound on dry ice. The PFA tissue was fixed O/N at 4 °C, followed by paraffin embedding using standard protocols and the blocks stored at room temperature for use in immunohistochemistry (IHC). The natively embedded material was transferred to −80 °C for use in cryo IHC. Analysis was performed for overall survival (OS) based on the total time the animal survived post-infection and for progression-free survival (PFS) based on time post-infection until the first symptoms indicated by a clinical score of 1.

### Statistical analysis

Statistics were performed using Prism 5.0/6.0 software (GraphPad, USA). A *p* value < 0.05 was considered significant. Data in graphs are represented as mean ± SEM with *p < 0.05; **p < 0.01; ***p < 0.001. The number of animals and experimental repeats are indicated in the corresponding figure legends including the statistical test employed.

## Results

### HIF-1α activation in neural cells in mouse and human pneumococcal meningitis

**Fig. 1:**
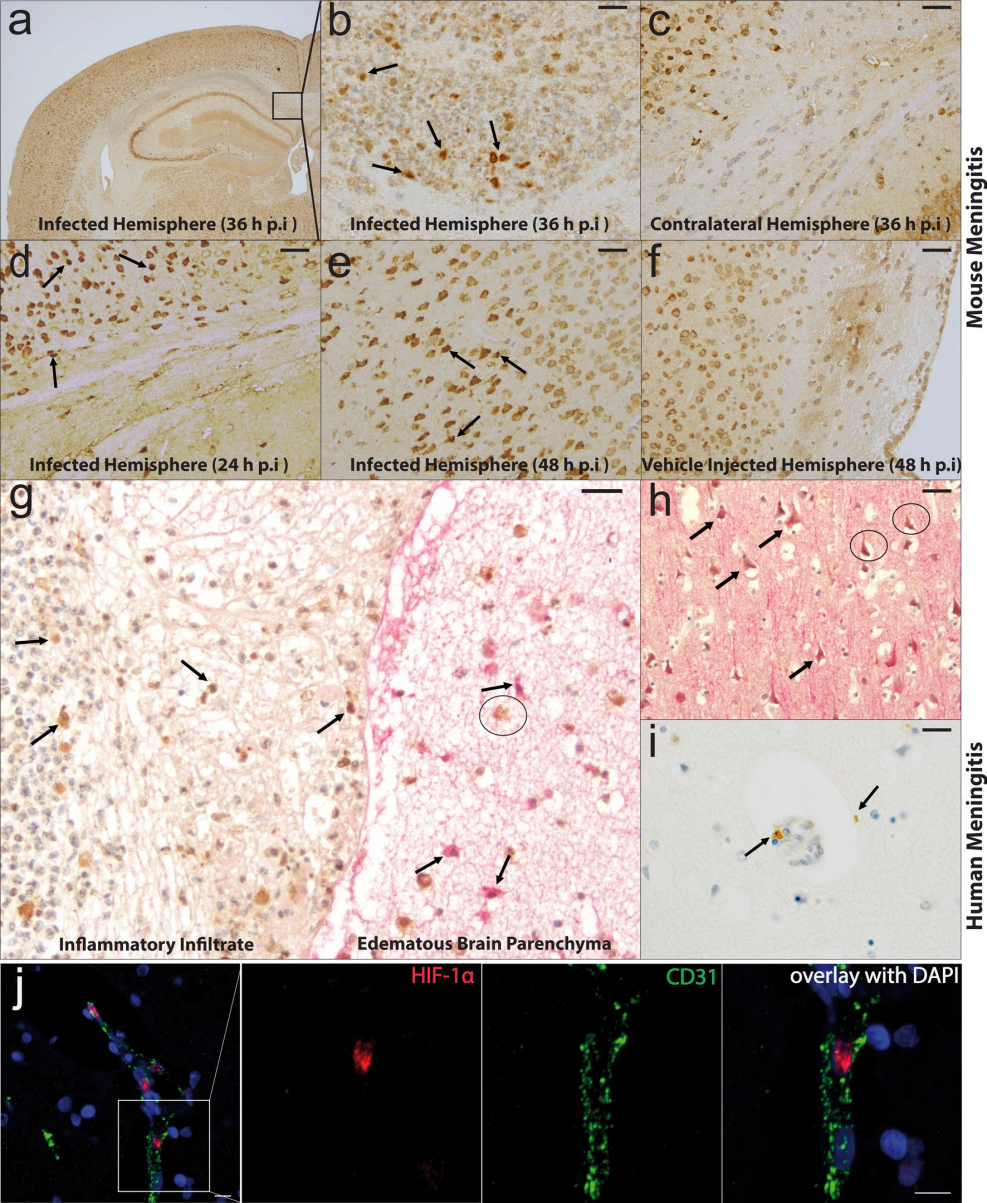
Induction of HIF-1α in mouse and human brain tissue samples from pneumococcal meningitis. **a** Immunohistochemistry of brain tissue 36 h post-*S. pneumoniae* infection in intracerebrally infected mouse model shows positive HIF-1α in several inflammatory and neural cells. **b** Zoomed area from **a** in the cortex region of the infected mouse brain shows significant nuclear HIF-1α (black arrows) indicating its activation along with positive staining in the granulocytic infiltrate. **c** Non-infected hemisphere of the same mouse at 36 h post infection shows no specific staining for HIF-1α signal. **d** Positive staining in the cortex region (black arrows) was also observed at 24 h post-intracerebral infection, which was also observed at **e** 48 h post-infection in the intracisternally infected mice. **f** Control mice that were intracerebrally injected with 0.9% NaCl did not show any positive staining in the same region. **g** Brain sections from human pneumococcal meningitis patients double stained for HIF-1α (brown) and GFAP (red) show nuclear staining for HIF-1α (black arrows) in lymphocytes of the inflammatory infiltrate (**g**, left) and in the edematous brain tissue comprising GFAP positive reactive astrocytes (**g**, right), where macrophages showed cytoplasmic staining (black circles). **h** Neurons labeled with MAP2 (red) were also co-stained with HIF-1α (brown) in the cortex but mostly the pyknotic ones and not the vital neurons (black circles). **i** Positive HIF-1α staining could also be observed in vessel associated cells in the cortex region. **j** Activated HIF-1α indicated by nuclear staining (red) could be observed in brains ECs from pneumococcal meningitis patients by immunofluorescence staining using CD31 as an endothelial marker. Scale bar **b**–**f** is 20 µm, **g**–**i** is 25 µm, **j **left most image 20 µm and the zoomed images 10 µm. Mouse brain stainings are representative of three mice each at 24, 36, 48 h post-infection and two control mice at 48 h post- vehicle injection. Human pneumococcal meningitis stainings are representative of 4 cases outlined in Table 1

HIF-1α activation is a general phenomenon upon infections [36, 75], and elevated VEGF levels known to be regulated via HIF-1α were found in CSF of meningitis patients [72]. To investigate the involvement of HIF-1α/VEGF pathway in the course of pneumococcal meningitis, we analyzed mouse and human brain specimen by HIF-1α immunohistochemistry (IHC). Murine pneumococcal meningitis was induced by intracerebral or intracisternal injection of *S. pneumoniae* (0.9% sterile NaCl for control animals) as described previously [32, 42]. By 24 h post-inoculation, all mice presented clinical signs of infection such as hypothermia, reduced vigilance, impaired motor function and weight loss. Mice were sacrificed at different time points and the brain tissue was immediately fixed in PFA and embedded in paraffin. 36 h post-intracerebral infection, granulocyte infiltrates were detected by hematoxylin/eosin staining in the ventricle and the white matter of the infected hemisphere, but not in the contralateral uninfected hemisphere (data not shown). At this time point, staining for HIF-1α was positive in the white matter and the surrounding cortex region with the typical nuclear staining pattern that indicated HIF-1α activation in the neural cells in the intracerebrally infected mice (Fig. 1a, b). The contralateral hemisphere did not show any sign of infection and HIF-1α staining was mostly cytoplasmic, excluding HIF-1α activation (Fig. 1c). Nuclear HIF-1α was also visible at 24 h post-infection in the cortex region in the intracerebral model (Fig. 1d) that was also confirmed at 48 h post-infection in an intracisternal infection model (Fig. 1e, f), in which the control vehicle injected mice did not show any specific HIF-1α staining. Similar findings were observed in human pneumococcal meningitis samples (Fig. 1 g-j, Table 1**)**. Staining for HIF-1α showed nuclear signal in leukocytes of the inflammatory infiltrate layer and in some astrocytes of edematous brain parenchyma double labeled with HIF-1α/GFAP (Fig. 1g). Macrophages, however, showed non-specific cytoplasmic signal indicating no HIF-1α activation. MAP2-stained neurons were also positive for nuclear HIF-1α but mostly the pyknotic neurons (Fig. 1h), which was also observed in the cells associated with small vessels in the cortex region (Fig. 1i). Double immunofluorescence staining for HIF-1α with CD31 as an endothelial marker also showed positive nuclei in ECs from three independent cases, indicating HIF-1α activation in brain ECs upon pneumococcal meningitis (Fig. 1j, supplementary Fig. 1, online resource). Activation of HIF-1α was also observed in other cases of mouse and human meningitis resulting from infections with bacteria (*E. coli*, *Cryptococcus neoformans*, *Mycobacterium tuberculosis*), fungi and protozoa (*Toxoplasma gondii*) (supplementary Figs. 2, 3, online resource). All these data demonstrate HIF-1α activation in both inflammatory and resident CNS cells upon meningitis in mice and humans.

### Activation of HIF-1α in brain endothelial cells upon in vitro pneumococcal infection

**Fig. 2:**
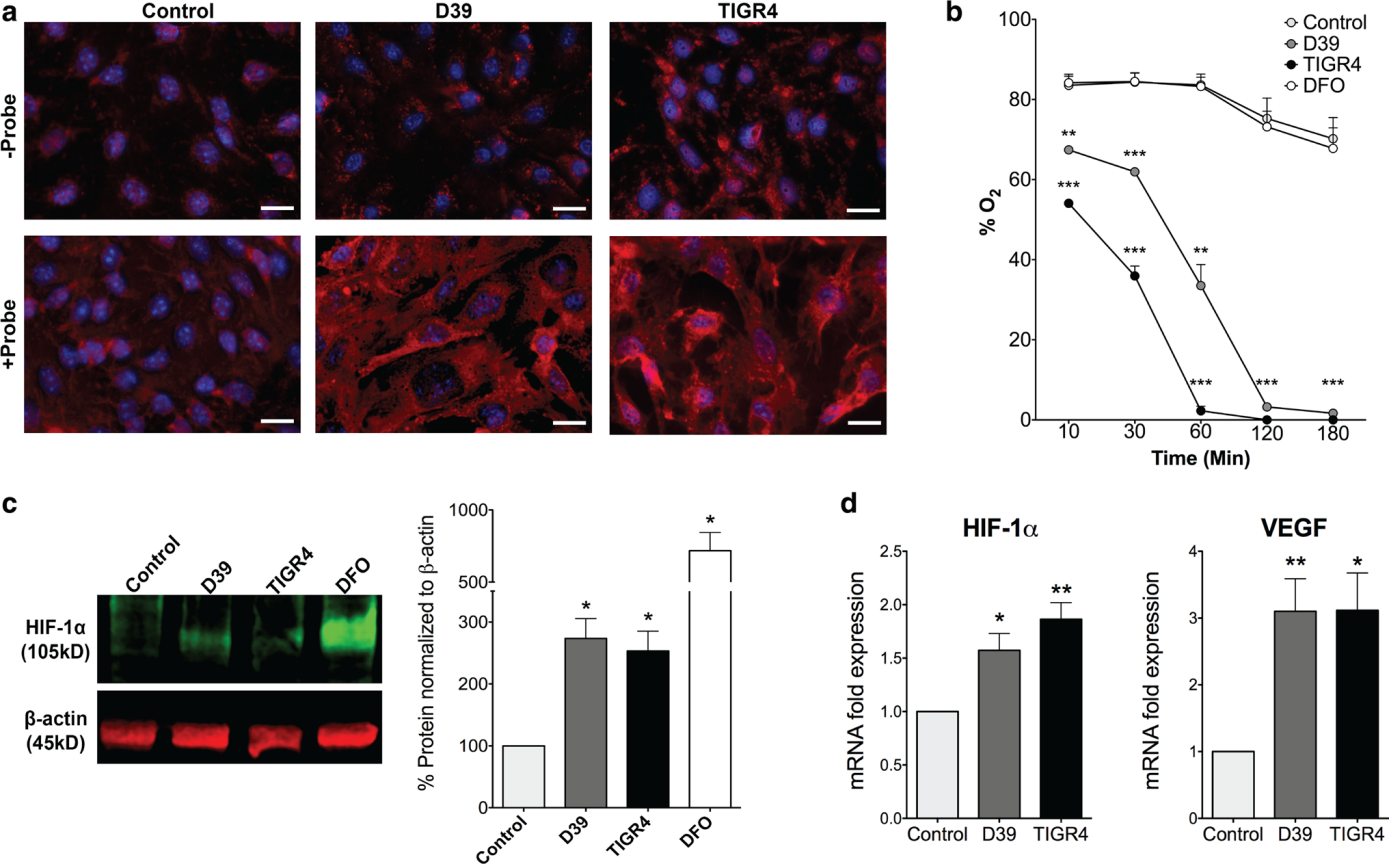
Hypoxia and HIF-1α/VEGF induction upon *S. pneumoniae* infection in brain ECs. **a** Control and *S. pneumoniae* infected bEnd5 cells treated with pimonidazole hydrochloride (Hypoxyprobe 200 mM) and visualized for cellular hypoxia by red 549-linked mouse anti-pimonidazole monoclonal antibodies. D39 and TIGR4 strains infected cells show significantly high number of hypoxic cells compared to control cells which is however not present in the top row without the probe indicating specificity of the staining (scale bar 10 µm). **b** Oxygen levels (%) were quantified in the medium of control and *S. pneumoniae* infected bEnd5 cells using the SensorDish® Reader (SDR) for non-invasive measurement of O_2_ levels through the transparent well bottom. O_2_ concentration (pO2% air saturation) was obtained at 60-s intervals and average values at several different time points shown indicate a significant reduction in O_2_ levels within minutes post-infection and to hypoxic levels by 2 h (*N* = 3, 2-tailed paired *t* test compared to control for each condition at the corresponding time point, mean ± SEM, **p < 0.01, ***p < 0.001), which was however not present either in control or deferoxamine (DFO, 200 µM), an iron chelator that induces HIF-1α independent of hypoxia. **c** Control and *S. pneumoniae* infected bEnd5 cells were analyzed for HIF-1α protein induction by Western blotting. The result shows a significant increase in HIF-1α levels in both D39 and TIGR4 infected cells compared to control conditions when the band pixel intensity is quantified by normalizing to the housekeeping protein β-actin band. DFO served as a positive control for HIF-1α induction (*N* = 4, *p < 0.05, 2-tailed paired *t* test compared to control for each condition). **d** Quantitative RT-PCR expression analysis (by 2^−∆∆Ct^ method) of control and infected bEnd5 cells shows upregulation of HIF-1α and its transcriptional target gene VEGF upon a *S. pneumoniae* infection. Expression was normalized to ribosomal protein, large, P0 (RPLP0) that served as a housekeeping gene (*N* = 6, *p < 0.05, **p < 0.01, 2-tailed paired* t *test compared to control)

It is known that the activation of the HIF-1α/VEGF pathway contributes to permeability of ECs [1, 25]. As HIF-1α activation was detectable in pneumococcal meningitis samples (Fig. 1), we hypothesized a critical role for this pathway in bacterial transfer across the BBB in infections. We infected brain ECs (immortalized brain endothelial cell line bEnd5) with two different *S. pneumoniae* strains (D39, TIGR4) in vitro to mimic bacterial interference at the BBB formed in vivo by ECs and co-regulated by pericytes and astrocytes. To investigate the mechanisms of *S. pneumoniae* transfer across BBB, which is an early event followed by bacterial replication in the parenchyma [68], we infected the brain ECs for a short duration of 2–6 h. Prolonged infection time caused a non-specific breakdown of the endothelial monolayer due to bacterial overgrowth and endothelial cell death. Induction of hypoxia known to trigger HIF-1α activation was analyzed by pimonidazole hydrochloride (Hydroxyprobe®, USA) staining and via direct quantification of oxygen consumption. Cellular morphology indicated dramatic effects on monolayer integrity upon infections, particularly for TIGR4. Infection resulted in a strong accumulation of pimonidazole indicating cellular hypoxia (Fig. 2a) and this was paralleled by a strong O_2_ decrease in *S. pneumoniae*-infected cell medium to below 2% after 3 h due to bacterial consumption (Fig. 2b). Congruently, hypoxic conditions were followed by HIF-1α activation as demonstrated by strongly elevated HIF-1α protein levels with both strains (~ three-fold induction, Fig. 2c) and via HIF-1α qRT-PCR (~ two-fold induction, Fig. 2d). Accordingly, VEGF, a confirmed target gene of the HIF-1α signaling cascade, was also induced in qRT-PCRs (~ three-fold induction, Fig. 2d). HIF-1α activation and VEGF induction were independent of the bacterial capsule (supplementary Fig. 4, online resource). Taken together, these results demonstrate a hypoxic HIF-1α/VEGF cascade in *S. pneumoniae* infections in vitro.

### Transmigration of bacteria and endothelial permeability upon *pneumococcal* infection in an in vitro BBB model

**Fig. 3:**
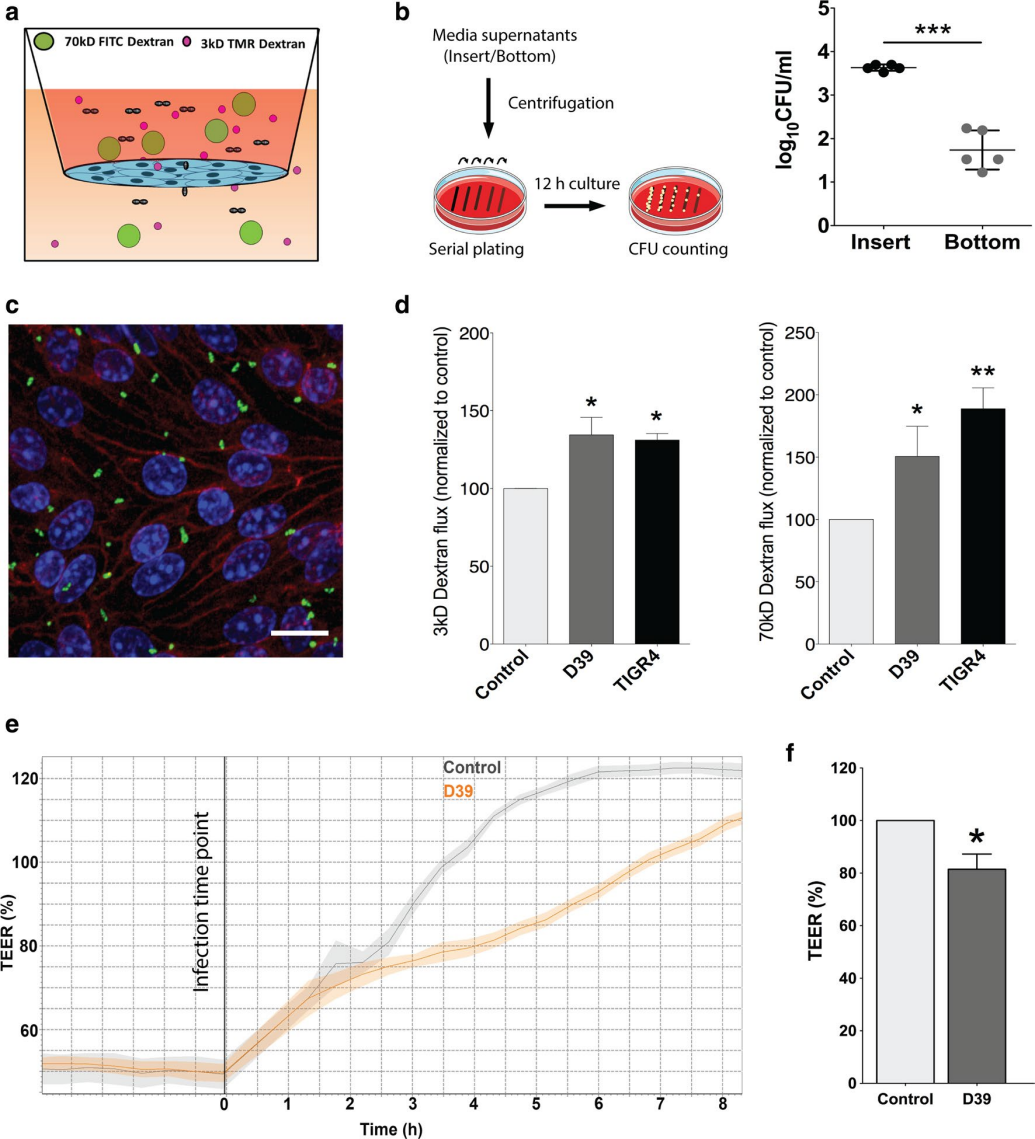
Bacterial transmigration and permeability upon *S. pneumoniae* infection in brain ECs. **a** The scheme depicts the in vitro model of the BBB for meningitis infection where bEnd5 cells were cultured in 24-well PET transwells (10^5^ cells/cm^2^) and infected with *S. pneumoniae* (D39 and TIGR4 strain; MOI 50) along with the addition of labeled dextrans to the apical chamber (5 μM each of 3 kD TMR, and 70 kD FITC dextran). **b** Media supernatant from infected and control cells from the insert and the bottom well at 4 h post-infection were collected and centrifuged for plating and culture on blood agar plate. The colony forming unit (CFU) assay indicates transmigration of approximately 1% of the bacteria added to the insert (*N* = 5, ***p < 0.001, 2-tailed paired *t *test compared to control). **c** Confocal microscopy analysis of transwell inserts to confirm the presence of bacteria indicated predominant localization of the bacteria (green) at the cell–cell borders with VE-cadherin (red) serving as EC junction marker (representative from *N* = 3 experiments; scale bar 10 μm). **d** Permeability analysis of bEnd5 cells 4 h post-infection performed with low (3 kD) and high molecular weight (70 kD) dextrans shows a significant increase in dextran permeability for both sizes, confirming the breakdown of endothelial barrier due to infection (*N* = 5, *p < 0.05, **p < 0.01, 2-tailed paired t-test compared to control). **e** Representative graph for continuous transendothelial electrical resistance (TEER) values of *S. pneumoniae* (D39) infected bEnd5 cells on inserts using the cellZscope® system setting the values of the control group between 3–4 h post-infection to 100%. The graph shows reduced TEER values starting 3 h post-infection that persists for several hours indicating increase in paracellular permeability. The increase in the TEER values of the control group from the plateau at 0 h reflects the changes due to electrode handling and media composition. **f** Quantification at 4 h shows a significant reduction in TEER values in infected cells compared to control (*N* = 4, *p < 0.05, 2-tailed paired *t* test)

As VEGF, a potent vascular permeability factor [1, 25], is induced by *S. pneumoniae* (Fig. 2), we assessed transmigration and localization of *S. pneumoniae* (D39 strain), followed by permeability analysis in an in vitro BBB model comprising brain ECs. For this, a transwell experimental setup was established with bEnd5 cells seeded on top of the insert (Fig. 3a). Infection was performed after 6 days when the cells formed confluent and tight monolayer mimicking a mature BBB in vitro. Infection from the apical side (top chamber) for 4 h led to the transmigration of ~ 1% bacteria across the EC monolayer into the bottom chamber (Fig. 3b). To confirm that the cells were not affected by bacterial overgrowth, the monolayers were stained for *S. pneumoniae* and ECs 4 h post-infection as described [8]. The endothelial marker VE-cadherin staining was junctional in all cells and their morphology appeared spindle shaped as expected for BBB ECs with no indication of gross cell layer disruption due to the infection (Fig. 3c). *S. pneumoniae* adhered to ECs predominantly at the cell–cell borders, suggesting a paracellular mode of transmigration across the ECs. We therefore performed paracellular permeability assays using fluorescently labeled dextrans of two different sizes (3 kD and 70 kD) to obtain size-specific permeability of ECs. Results showed a significant increase in the permeability of bEnd5 cells to both sizes of dextran upon infection with D39 or TIGR4 strains (Fig. 3d), which is supported by decrease in expression of junction molecules (supplementary Fig. 5, online resource). Continuous impedance measurements of ECs in the CellZscope device revealed reduced transendothelial electrical resistance (TEER) commencing 3 h post-infection that persisted up to 8 h (Fig. 3e, f). Increase in EC permeability was also present in infections with capsule-deficient mutants in line with induction of HIF-1α and VEGF by these strains (supplementary Fig. 4, online resource). Summarized, these data show that infection with *S. pneumoniae* results in bacterial translocation and a breakdown of barrier function in an in vitro BBB model

### Neurovascular permeability and CNS deposition of *S. pneumoniae* in a murine model of hematogenous infection

**Fig. 4:**
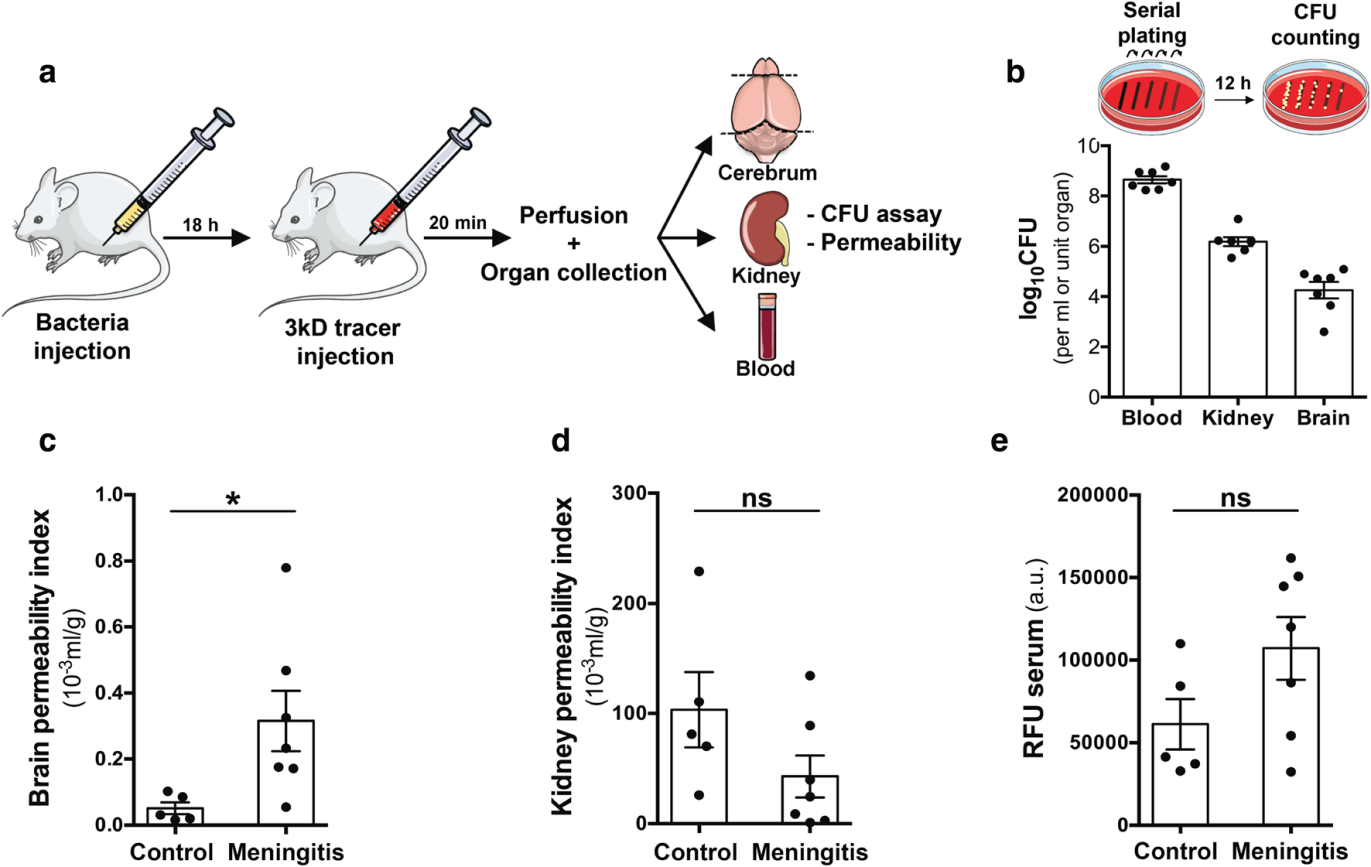
Extravasation of *S. pneumoniae* and neurovascular permeability in vivo in a hematogenous meningitis model. **a** Setup showing the murine infection model with intraperitoneal injection of 0.5 × 10^5^ bacteria (in 100 μl PBS, D39 strain). 18 h post-infection mice were injected with a 3kD TMR tracer (i.p), circulated for 20 min followed by anesthesia, transcardial PBS perfusion and collection of blood, brain, and kidney. **b** Homogenized organs or blood were plated and cultured overnight to obtain the bacterial counts using the CFU assay. High CFU values in blood indicated the hematogenous presence of bacteria with extravasation of bacteria evident in the brain (0.01%). The bacterial counts in the kidney were 100-fold higher than brain reflecting higher permeability of the kidney vasculature. Healthy control mice did not have any bacterial growth on the plates. **c** Homogenized hemi-brain, kidney and blood were utilized to obtain dextran permeability by measuring fluorescence intensity on a microplate reader. A significant elevation in vascular permeability of infected mice was observed compared to healthy controls in the brain indicating blood–brain barrier breakdown. **d** Vascular permeability of kidney was not altered upon infection. **e** Serum fluorescence values (arbitrary units—a.u.) indicate equivalent tracer absorption between healthy and infected mice. (*N* = 5–7/group, *p < 0.05, 2-tailed unpaired *t* test)

As a hematogenous infection model is most appropriate for bacterial transmigration studies across BBB, we infected mice intraperitoneally with *S. pneumoniae* and assessed the course of infection by plating of infected blood and subsequent CFU determination (Fig. 4a). In the infected mice, we detected a significant amount of bacteria deposited in the brain compared to blood levels revealing a successful transmigration process of *S. pneumoniae* over the BBB or the blood–CSF barrier (Fig. 4b). Peripheral infection was confirmed with even greater level of bacteria in the kidneys where the vascular barrier is leakier compared to the BBB. Permeability analysis of the injected 3 kD dextran tracer indicated a higher dextran levels in the brain of infected animals compared to healthy controls, whereas the kidney levels of the tracer were in the same range but higher compared to the brain levels, indicating a greater basal permeability of the kidney vasculature (Fig. 4c, d). Similar serum levels confirmed the presence of injected tracer in all the animals analyzed (Fig. 4e). The increase in BBB permeability in vivo upon infection could also result from dysfunction of other NVU cells (supplementary Fig. 6, online resource). The increase in EC permeability upon *S. pneumoniae* infection both in vitro and in vivo and the localization of *S. pneumoniae* in bEnd5 cells at the cell–cell junctions suggest a paracellular route of bacterial transmigration across the BBB.

### Paracellular localization and transmigration of *S. pneumoniae* across brain ECs

**Fig. 5:**
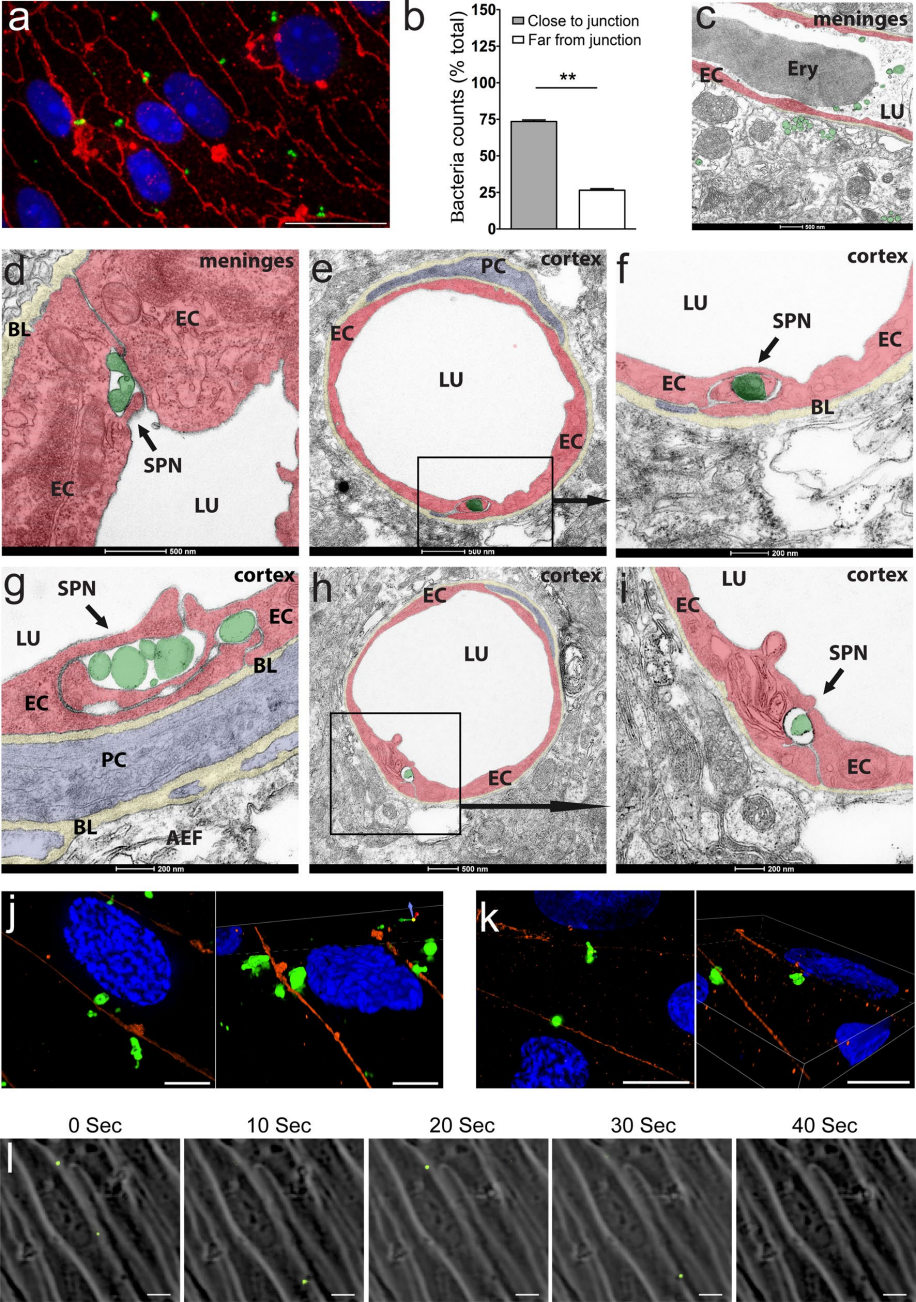
Junctional localization of *S. pneumoniae* at the BBB in vitro and in vivo. **a** Primary mouse brain ECs (MBMEC) were infected with *S. pneumoniae* and subjected to immunofluorescence staining for claudin-5 to mark endothelial junctions (red) and co-stained with an anti-pneumococcal antibody (green) to label *S. pneumoniae*. Confocal fluorescence microscopy of EC monolayers indicated a predominant localization of bacteria at the cell–cell borders co-localizing with claudin-5, an endothelial tight junctions marker as shown by **b** quantification that indicated a significantly higher number of bacteria close to the junctions. *N* = 3 independent preparations of MBMEC from 2–3 mice each time. Two independent wells were counted for each set comprising approximately 25 cells/field at 60X magnification (scale bar: 20 μm). Bacteria within a distance of 1 bacterium equivalent size from the junction were considered close to the junction. Data presented as mean ± SEM, **p < 0.01, 2-tailed paired *t* test. **c–i** Transmission electron microscopy (TEM) analysis was performed on brain sections from hematogenously infected mice post-perfusion with PBS/PFA and fixation in PFA/glutaraldehyde. *S. pneumoniae* either directly or in protective membrane bound vesicles were localized at the endothelial tight junctions both in meninges and cortex in several mice analyzed (representative images from *N* = 6 mice subjected to TEM). Artificial coloration was performed for better visualization and to highlight the localization of *S. pneumoniae*. SPN-*S. pneumoniae*, EC-endothelial cell, PC-pericyte, LU-lumen, BL-basal lamina, AEF-astrocytic endfeet, ERY-erythrocyte. **j–k** Primary human brain ECs (HBMEC) were also infected with *S. pneumoniae* and stained with anti-pneumococcal antibody and claudin-5 and subjected to super resolution microscopy using Nikon structured illumination microscopy (N-SIM). In 2 different preparations of HBMEC, the localization of bacteria was primarily at the junctions with super resolution images demonstrating engagement of *S. pneumoniae* with the endothelial junctions (Figures to the right both in **j, k**). Scale bar: 5 μm. **l** Primary mouse brain ECs (MBMEC) were infected with GFP-labeled *S. pneumoniae* strain (MOI 10) 2 days post-isolation and subjected to live-cell imaging. MBMECs as well as bacteria were imaged in brightfield and GFP fluorescence channel every 10 s starting 1 h post-infection for a total of 2 h. Five time-lapse images (10-s interval) capture transmigration of few bacteria across cell–cell borders (whites lines), which demonstrate paracellular route for transmigration of *S. pneumoniae* across brain endothelial cells. Representative images from 1 preparation from *N* = 3 MBMECs preparations (1 animal/set) infected with GFP-labeled D39 strain. Scale bar: 10 μm.

To confirm the localization of *S. pneumoniae* observed in bEnd5 cells (Fig. 3c), we performed confocal analysis of infected primary brain ECs isolated from wild-type mice and quantified the number of bacteria close to cell junctions. *S. pneumoniae* adhered to primary ECs also at the cell–cell borders and their quantification indicated a predominant localization close to the cell–cell junctions (Fig. 5a, b). To confirm these observations in vivo, electron microscopy was performed on hematogenously infected mice. Meningeal infection was confirmed by the presence of extravascular bacteria in the meninges (Fig. 5c, bacteria colored green). Both in meninges and cortex regions (Fig. 5d–i), *S. pneumoniae* localization was at the cell–cell junctions, which was verified in several infected mice (supplementary Fig. 7, online resource). To test whether this localization also appears in human BBB, primary human brain ECs were infected with *S. pneumoniae* and subjected to super-resolution microscopy (SRM). Bacterial localization was again predominantly at the cell–cell junctions (Fig. 5j, k). To unequivocally demonstrate the paracellular preference of *S. pneumoniae* in transmigration across brain ECs, we performed live-cell imaging analysis of primary mouse brain ECs infected with these bacteria. At 2 days post-isolation, MBMEC were infected (MOI 10) with GFP-labeled D39 *S. pneumoniae* strain [40], followed by live-cell imaging. Within 2 h post-infection, several bacteria transmigrated across cell–cell borders with a duration of 30–45 s to transmigrate once localized in the paracellular space, as observed in the time lapse images and video (Fig. 5l and supplementary video 1, online resource). Taken together, these data demonstrate a paracellular route for transmigration of *S. pneumoniae* across brain ECs at the BBB.

### Regulation of HIF-1α/VEGF signaling at the BBB in murine pneumococcal meningitis

**Fig. 6:**
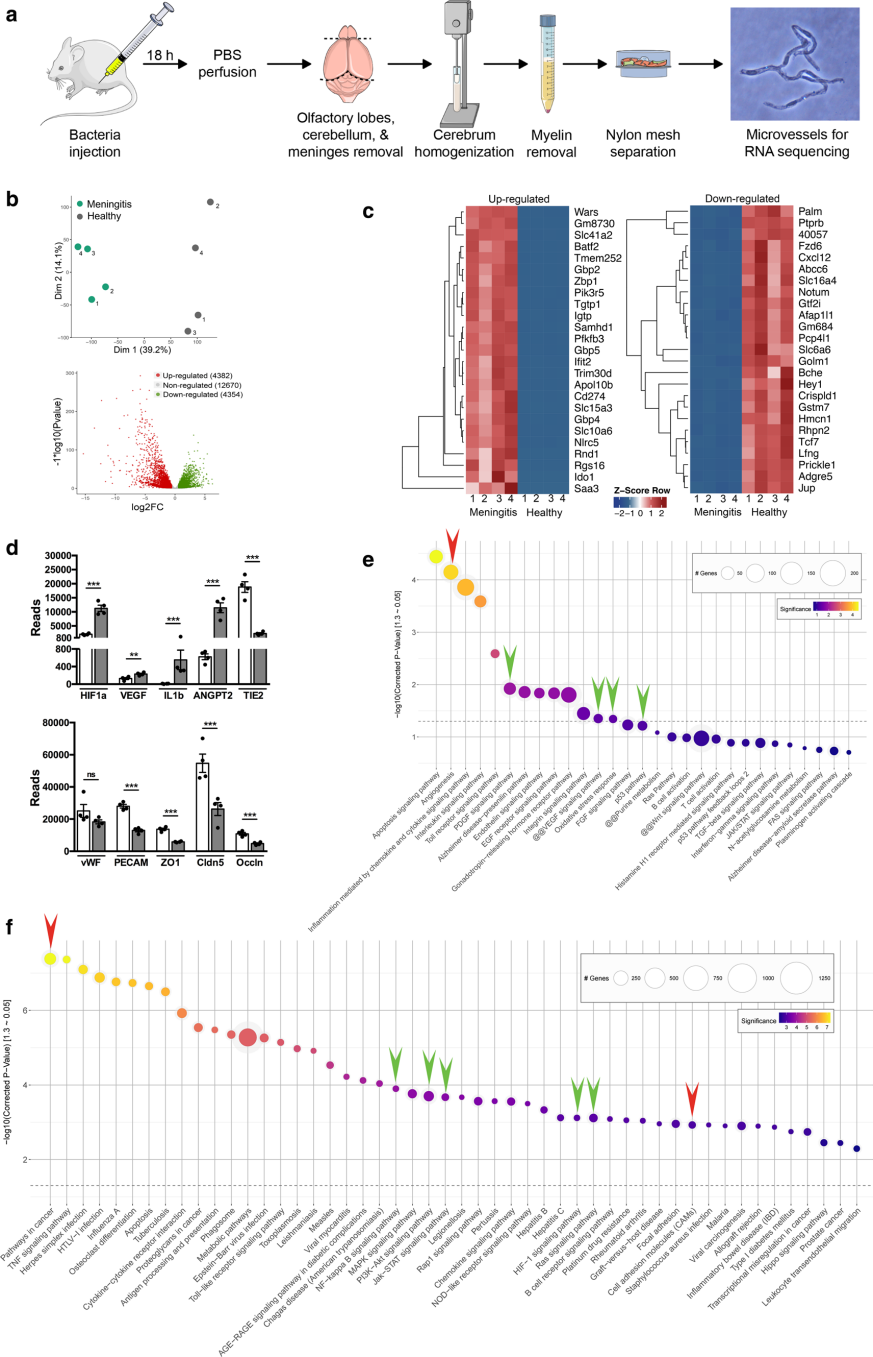
Regulation of HIF-1α/VEGF signaling by RNAseq analysis of brain microvessels from *S. pneumoniae* infected mice. **a** Schematic of BBB microvessels isolation for RNAseq analysis showing intraperitoneal injection of bacteria followed by anesthesia and transcardial perfusion 18 h post-infection. Extracted brains were cleared of meninges, olfactory lobes, and cerebellum and homogenized followed by myelin removal (density centrifugation). The microvessels were separated by filtering through 100 μm nylon mesh and collected from the top of 40 μm mesh. **b** The PCA plot shows clear clustering of healthy and meningitis microvessels with the volcano plot showing close to 9000 differentially expressed genes (DEG). **c** Heatmap analysis shows top 25 upregulated and downregulated genes by Z-score transformation. The data are displayed in a grid where each row represents a gene and each column represents the sample replicate. **d** Visualization of actual base reads of gene expression by RNAseq profiling shows upregulation of HIF-1α, VEGF and inflammation-related genes such as IL1b and ANGPT2. BBB junction molecules claudin-5, occludin, ZO-1 were downregulated, indicating breakdown of the BBB in this mouse model at the transcriptomic level. **e** PANTHER pathway analysis of the RNAseq data illustrates the activation of pathways related to angiogenesis (red arrow) and HIF-1α/VEGF signaling, and the ones related to them such as PDGF, p53 pathways (green arrows). **f** The KEGG pathway enrichment also indicated activation of HIF-1α/VEGF signaling, and related pathways driven by NF-kappa B, PI3-Akt, Jak-STAT, and Ras signaling (green arrows). Pathways related to cancer and vascular remodeling were also activated (red arrows) including those related to inflammation. Dotted line in **e, f** indicates the cutoff value for significance with color coding for significance level (lighter being more significant) and bubble size reflecting the number of genes in the significant hits (*N* = 4 samples/group pooling 2 brains per sample)

The BBB is the critical interface in the initiation of meningitis. We therefore analyzed gene regulation and signaling pathways in the BBB forming microvessels. Mice were intraperitoneally infected and brain microvessels were isolated for RNA sequencing and bioinformatic analyses as described previously [37, 16] comparing them with healthy controls (Fig. 6a). The dataset has been uploaded to NCBI GEO database (ID: GSE122952). As observed via principal component analysis (PCA) and the corresponding volcano plots, gene expression at the BBB in meningitis is significantly altered with ~ 20% (4,382 genes) genes upregulated and ~ 20% (4,354) downregulated; the complete hierarchical clustering data are included (supplementary Fig. 8, online resource). The top 25 regulated genes (based on significance) are shown in the heatmaps (Fig. 6b, c). Also, in this in vivo murine pneumococcal meningitis model, we observed an upregulation of HIF-1α and VEGF at the BBB (Fig. 6d). Several genes known to be regulated by HIF-1α, such as angiopoietin2, Tie2, PTPRB that we reported previously for their role in BBB function in ischemic stroke and brain tumors [27, 37, 16], were dysregulated in the infected mice. Additionally, several BBB junctional genes such as occludin, claudin-5, and ZO-1 were found to be downregulated indicating a BBB breakdown (Fig. 6d). Several proinflammatory cytokines such as IL1β and TNFα were also induced, indicating the inflammatory status of the mice at the BBB level. Bioinformatic analyses using KOBAS indicated the activation of HIF-1α/VEGF pathway independently by pathway enrichment analysis in PANTHER and KEGG databases (see green arrows, Fig. 6e, f) and also in Reactome database analysis (supplementary Fig. 9, online resource). Several related pathways such as NF-kappa B, PI3K-Akt, Jak-STAT, Ras and PDGF were also activated at the BBB upon pneumococcal infection. Pathways related to vascular remodeling and angiogenesis were also activated indicating a gross BBB dysfunction in meningitis (red arrows in Fig. 6e, f). These data strongly support the involvement of HIF-1α/VEGF pathway at the BBB in vivo in pneumococcal meningitis.

### HIF-1α dependence of BBB permeability in *S. pneumoniae* infections

**Fig. 7:**
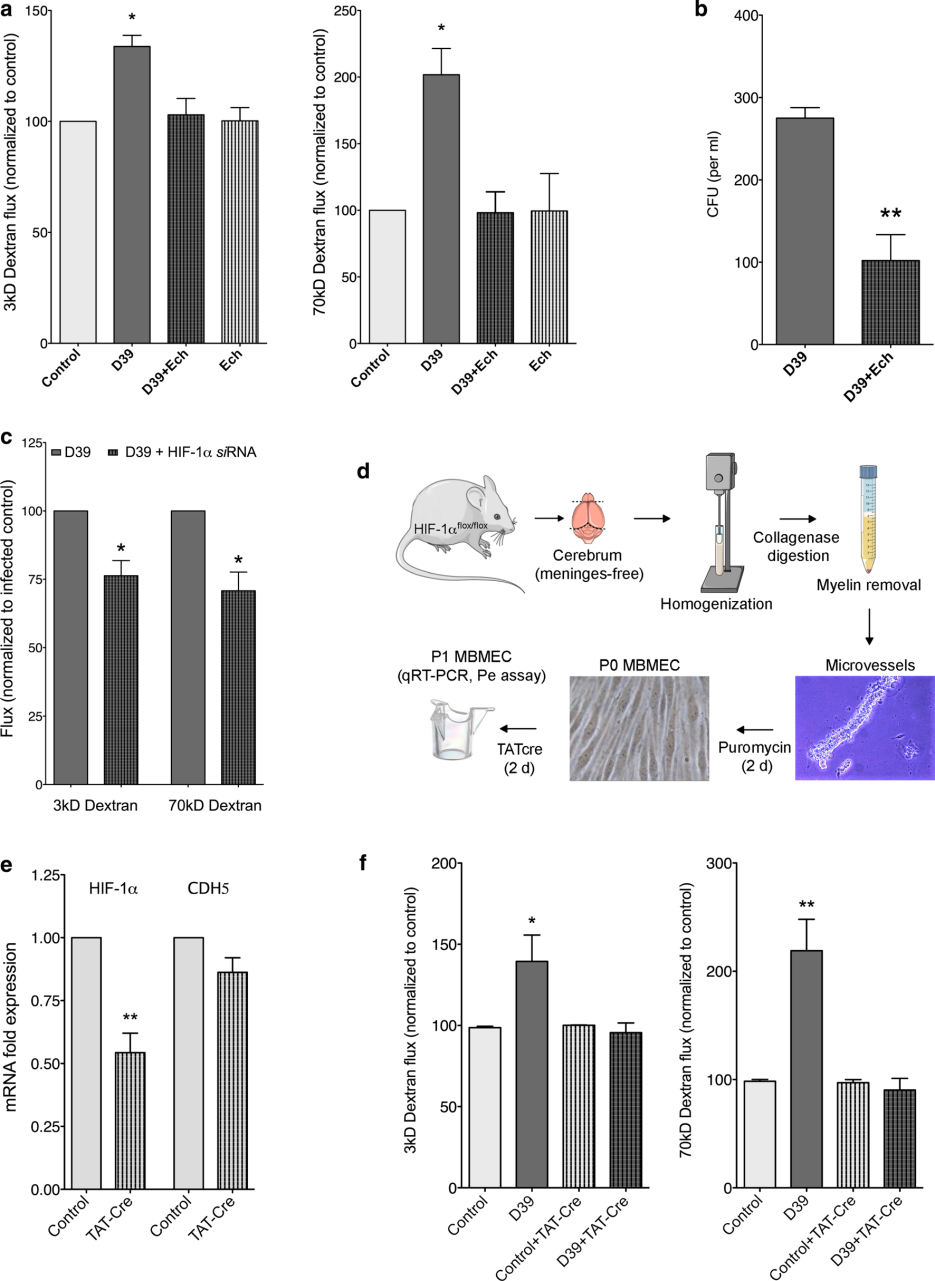
HIF-1α-dependent endothelial permeability and transmigration of *S. pneumoniae* post-infection. **a** Treatment of brain ECs (bEnd5) with echinomycin (Ech), an HIF-1α inhibitor, did not lead to higher permeability post-*S. pneumoniae* infection (D39 strain) for both low (3 kD) and high molecular weight (70kD) dextrans, whereas untreated cells showed increase for both the tracers. Ech alone did not affect permeability when compared to uninfected controls. (mean ± SEM, *N* = 3, *p < 0.05, 2-tailed paired *t* test. **b** Ech treatment also led to significant reduction in the transmigrated bacteria across bEnd5 monolayers, which combined with Fig. 1a indicates HIF-1α dependence of both permeability and paracellular bacterial transmigration (mean ± SEM, *N* = 3, **p < 0.01, 2-tailed unpaired *t* test). **c** Genetic ablation of HIF-1α using *si*RNA-mediated knockdown in bEnd5 cells also demonstrated a decrease in permeability post-infection compared to scrambled controls (mean ± SEM, *N* = 4, *p < 0.05, 2-tailed paired *t* test). **d** Schematic of primary mouse brain endothelial cells (MBMEC) isolation and culture for permeability analysis post infection. Cerebral hemispheres from HIF-1α^flox/flox^ mice cleared of meninges were homogenized followed by myelin removal and collagenase digestion. Plated microvessels were treated with puromycin to obtain pure EC cultures, followed by treatment with TATcre to induce knockdown of HIF-1α and seeded on to transwell inserts for infection and permeability analysis. **e** Quantitative RT-PCR confirmed about 50% knockdown of HIF-1α in MBMEC when normalized to RPLP0 (a housekeeping gene), VE-cadherin (CDH5) was not changed (mean ± SEM, *N* = 5 MBMEC preparations pooling 2–3 mice/set, **p < 0.01, 2-tailed paired *t *test). **f** Permeability analysis of MBMEC to both low and high molecular weight dextrans showed an increase post infection with *S. pneumoniae* (D39) as observed with bEnd5 cells. This effect was abrogated upon HIF-1α knockdown in TAT-cre treated cells demonstrating HIF-1α dependence of permeability post infection also in primary brain endothelium (mean ± SEM, *N* = 6 MBMEC preparations pooling 2–3 mice/set, *p < 0.05, **p < 0.01, 2-tailed paired *t* test)

We demonstrated that *S. pneumoniae* infection in vitro and in vivo induced expression of HIF-1α and its target gene, VEGF in the brain endothelial cells and also leads to increased permeability (see above). However, as VEGF is known to induce paracellular permeability in brain ECs [1] and given the paracellular localization of *S. pneumoniae* in these cells, we investigated whether the permeability upon bacterial infection is mediated by HIF-1α/VEGF pathway. To this end, we first utilized echinomycin, a potent chemical inhibitor of HIF-1α [43]. In bEnd5 cells, echinomycin treatment followed by *S. pneumoniae* infection prevented EC permeability to low or high molecular weight dextrans when compared to untreated controls (Fig. 7a). Furthermore, echinomycin treatment reduced bacterial transmigration into the bottom chamber across the endothelial monolayer (Fig. 7b). Similar effects on permeability were also observed in bEnd5 cells upon *si*RNA-mediated knockdown of HIF-1α (Fig. 7c), the efficiency of which was verified by qRT-PCR (not shown). To confirm these results, we performed infections in primary brain ECs. However, as primary brain ECs are not amenable to transfection, we utilized a mouse model that is homozygous for a floxed allele of HIF-1α at the corresponding locus (HIF-1α^flox/flox^, [62]). Brain ECs were isolated from HIF-1α^flox/flox^ mice as shown in the schematic (Fig. 7d), followed by treatment with TAT-Cre, a cell-permeable cre-recombinase for in vitro recombination of loxP sites to knock out HIF-1α in isolated cells. Treatment of brain ECs resulted in a ~ 50% knockdown of HIF-1α as shown by qRT-PCR (Fig. 7e). HIF-1α knockdown in these primary brain ECs resulted in a reduction of permeability (3 kD and 70 kD dextrans) back to the level of uninfected cells (Fig. 7f). Similar results were also obtained in human brain ECs by *si*RNA-mediated knockdown of HIF-1α (supplementary Fig. 10, online resource**).** These data demonstrate that *S. pneumoniae*-induced permeability is dependent on HIF-1α/VEGF pathway.

### Therapeutic rescue of murine *S. pneumoniae* infection by echinomycin to inhibit HIF-1α

**Fig. 8:**
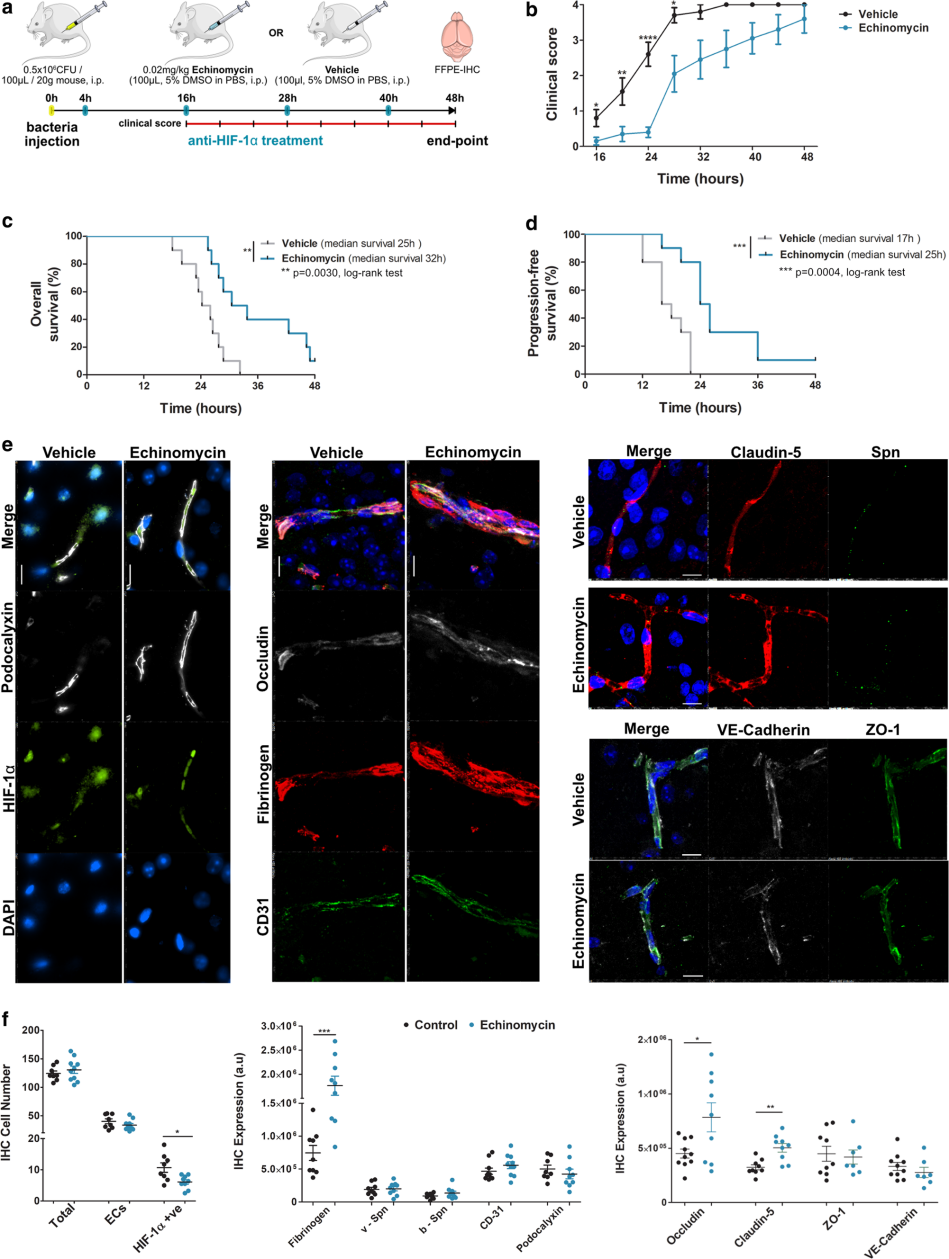
Improved survival and blood–brain barrier function post pneumococcal infection in mice by anti-HIF-1α treatment using echinomycin. **a** Schematic depicting treatment protocol of mice infected with *S. pneumoniae* (D39). Mice were infected i.p., followed by anti-HIF-1α treatment with echinomycin or just vehicle every 12 h starting 4 h post-infection (blue tick marks). Clinical scores were taken every 4 h starting 16 h post-infection (red line with tick marks) and study terminated at 48 h post-infection and brain tissues collected upon animal death or at the end point when the animals were sacrificed. **b** Clinical scores indicate improved symptoms in echinomycin-treated mice compared to vehicle-treated animals starting 16 h post-infection that were progression free (below score 1) up to 24 h (mean ± SEM at each time, *N *= 10/group, ****p < 0.0001, **p < 0.01, *p < 0.05 by 2-tailed unpaired non-parametric Mann–Whitney *t* test at each time point. **c** Echinomycin-treated mice showed significantly improved overall survival percentage by Kaplan–Meier analysis with the median survival at 32 h compared to 25 h in vehicle-treated mice (**p < 0.01, log-rank test, *N* = 10/group). **d** Kaplan–Meier analysis also indicated a significant improvement in progression-free survival in echinomycin-treated animals (median survival 25 h) with no clinical symptoms up to 24 h (**b**) compared to the vehicle group (median survival 17 h; ***p < 0.001, log-rank test, *N *= 10/group). **e** Immunofluorescence staining for HIF-1α, BBB permeability and junctional markers in the echinomycin and vehicle groups at the survival end point. Left panel shows that echinomycin treatment leads to a reduction in HIF-1α-positive nuclei including in ECs co-stained for podocalyxin, a vascular marker which was unchanged by the treatment. Middle panel displays reduced vascular permeability to fibrinogen in the echinomycin group as indicated by stronger intravascular signal compared to the vehicle-treated mice. Increased expression of tight junction proteins—occludin (middle panel) and claudin-5 (right top panel)—in echinomycin-treated mice indicate improved BBB function. Tight junction-associated ZO-1, adherens junction marker VE-Cadherin, and endothelial cell adhesion molecule CD31 were unchanged (middle, bottom right panel). There was also no difference in *S. pneumoniae* (Spn) staining (right top panel) between the two groups. Scale bar 10 μm. **f** Quantification of the staining from **e** utilizing four images per animal in the cortex region shows a significant reduction in HIF-1α-positive cell number, whereas the total cell number or EC cell number was unchanged (left panel). As observed in **e**, the middle panel for quantification (arbitrary units—a.u.) of fibrinogen leakage shows significantly increased vascular staining for fibrinogen, supported by significantly increased expression of occludin, claudin-5, whereas other EC junction markers were unchanged. *S. pneumoniae* numbers in the vessels (v-Spn) or those transmigrated into brain parenchyma (b-Spn) were also unchanged. (mean ± SEM, *N* = 7–10/group indicated by the corresponding number of dots, ****p < 0.0001, **p < 0.01, *p < 0.05 by 2-tailed unpaired Student’s *t *test)

Echinomycin, a potent small molecule inhibitor of HIF-1α, was used as anti-HIF-1α treatment to therapeutically rescue mice infected with *S. pneumoniae*. Adult wild-type mice were intraperitoneally infected with *S. pneumoniae* as described previously, followed by treatment with echinomycin every 12 h starting 4 h post-infection. This survival study was conducted for 48 h with clinical scoring every 4 h starting 16 h post-infection. The experimental design is shown in Fig. 8a. Compared to the vehicle-treated mice, the echinomycin-treated mice showed significantly improved clinical scores at several time points starting 16 h post-infection which was maintained up to 28 h (Fig. 8b). Both, the overall survival and progression-free survival, as anticipated from improved clinical scores, were significantly improved in the treated animals suggesting a therapeutic rescue of pneumococcal infection with echinomycin (Fig. 8c, d). Immunohistochemistry (IHC) at the end point revealed significant reduction in HIF-1α-positive cells including brain ECs (co-stained for podocalyxin, a vessel marker) in the group treated with echinomycin, indicating it is specific in HIF-1α inhibition (left panel in both Fig. 8e, f). Analysis of BBB function indicated reduced extravasation of fibrinogen from the brain vessels of echinomycin-treated animals compared to the vehicle group (middle panel in Fig. 8e, f). This was supported by the increased expression of tight junction markers occludin and claudin-5 in the echinomycin group compared to the vehicle group (Fig. 8e middle and right-top panels and quantified in Fig. 8f right panel). However, EC adhesion molecule CD-31, vascular glycocalyx member podocalyxin, adherens junction member VE-cadherin, and tight junction-associated molecule ZO-1 were all unchanged between the two groups (Fig. 8e, f). Interestingly, *S. pneumoniae* staining in the vessels (v-Spn) and in brain parenchyma (b-Spn) was unchanged between the groups (Fig. 8e right top, quantified in Fig. 8f middle panel), suggesting an absence of bactericidal effects of echinomycin in this murine model. Comparison to control healthy animals by immunohistochemistry confirmed the improved BBB function by echinomycin compared to the vehicle (supplementary Fig. 11, online resource). Overall, these data demonstrate improvement of BBB function by HIF-1α inhibition with echinomycin, leading to improved clinical symptoms and survival post-pneumococcal infections.

## Discussion

The homeostasis of the CNS is maintained by the BBB, which is formed by brain ECs and co-regulated by other cells of the NVU. The tight junctions between the ECs and the efflux transporters expressed on their plasma membranes limit the transport of blood-borne solutes and pathogens [46]. In bacterial meningitis, the circulating bacteria first enter the CNS via the BBB or the blood–CSF barrier (BCSFB), consequently leading to infection and the ensuing neurological complications, mainly cerebral edema and elevated intracranial pressure. The most frequent pathogen causing bacterial meningitis is *S. pneumoniae* that commonly resides in the mucosal layers of the nasopharynx, but occasionally penetrates the tissue barriers, leading to invasion and survival in the blood stream. Meningitis results when the blood-borne pathogens translocate across the BBB and cause CNS infection [18]. Bacterial transmigration across the BBB is therefore a critical step in the course of disease.

Involvement of endothelial β2-adrenoreceptor has been identified in transmigration of meningococcal pathogens across the BBB [7]. Bacterial induction of Snail in brain ECs has been reported in group B streptococci (GBS) meningitis, leading to disruption of the BBB and penetration of meningeal pathogens [39]. Interaction of *S. pneumoniae* with platelet activating factor receptor (PAFR) [33, 60] and also with polymeric Ig receptor and PECAM was demonstrated to be important for bacterial adhesion including for brain endothelium [34]. However, the exact mechanisms in the host endothelium responsible for bacterial transmigration and their route of transmigration are still poorly understood in pneumococcal meningitis [18, 33, 38].

We have previously reported that HIF-1α activation and the induction of its target gene VEGF [28, 75] is a general phenomenon in a broad variety of infections due to bacteria, fungi and viruses and characterized this phenomenon in more detail in *Bartonella henselae* infections, leading to vasculoproliferative disorders [35, 36, 59]. Increased VEGF levels were also reported in inflammatory infiltrates of bacterial meningitis autopsy specimen [72]. Furthermore, oxidative stress due to excessive production of reactive oxygen species (ROS) has been reported in several studies during pneumococcal meningitis, again suggesting a potential involvement of HIF-1α, as ROS is a well-known HIF-1α activator [14, 41]. Given the established role of HIF-1α/VEGF signaling in endothelial permeability [1, 23, 25], we hypothesized a critical role for this pathway in the BBB permeability and bacterial transmigration in meningitis. Immunohistochemistry of murine pneumococcal meningitis samples revealed activated HIF-1α in the brain and this finding was confirmed in human pneumococcal and other bacterial meningitis autopsy specimen. In brain ECs (bEnd5 cells), pneumococcal infection with clinical *S. pneumoniae* isolates (D39, TIGR4) led to dramatic reduction of oxygen levels within minutes of infection, leading to hypoxia that persisted for hours resulting in HIF-1α activation at RNA and protein levels in brain ECs. Furthermore, the infections led to an induction of VEGF, a known target gene of HIF-1α transcriptional activity. As VEGF causes paracellular permeability in ECs [1, 25], we utilized a transwell setup with apical infection of bEnd5 monolayers to assess permeability. Deposition of bacteria in the basal chamber and their predominant localization at the cell–cell borders suggest a paracellular transmigration of bacteria. Permeability analysis using different sizes of dextran showed an increase, which is reflective of paracellular permeability as brain ECs undergoes minimal fluid-phase transcytosis. These data are also supported by reduced TEER values upon infection obtained from continuous impedance measurements [8]. To elaborate the mechanisms of bacterial transmigration across the brain endothelium in vivo, an intraperitoneal infection model was used where pneumococci are distributed hematogenously with deposition in the CNS. When analyzing permeability and bacterial localization (via electron microscopy, super-resolution microscopy and confocal microscopy), we observed increased vascular permeability and localization of bacteria at the cell–cell junctions in brain ECs. Paracellular transfer of *S. pneumoniae* was also observed by live-cell imaging of primary brain ECs infected with of GFP-labeled bacteria. From this data, we conclude that *S. pneumoniae* utilize paracellular pathway for transmigration across the BBB in meningitis.

As our data argue for an involvement of HIF-1α/VEGF from both in vitro infection experiments and from immunohistochemical analysis of meningitis samples, we elaborated HIF-1α/VEGF expression in vivo at the BBB. To this end, brain microvessels were freshly isolated post-infection and RNA sequencing was performed to obtain the transcriptional changes in an unbiased manner as we described previously [37, 16]. Hematogenous infection of *S. pneumoniae* resulted in global transcriptional changes in the BBB microvessels demonstrated by the regulation of ~ 9000 genes out of ~ 21,000 genes. Several BBB junction molecules were found to be downregulated in meningitis including occludin, claudin-5 and ZO-1, highlighting the BBB breakdown. Several proinflammatory cytokines were found to be upregulated such as IL-1β and TNF-α, supporting increased inflammation as observed in bacterial meningitis cases [57, 68]. Further analysis indicated a significant upregulation of HIF-1α and VEGF and these findings were supported by bioinformatics pathway analyses, showing increased HIF-1α/VEGF signaling at the BBB in meningitis. Junctional localization of bacteria and increased paracellular permeability upon infection establish a paracellular pathway for bacterial transmigration at the BBB. This is further supported by downregulation of junctional molecules, unchanged transcellular permeability factor plasmalemma vesicle associated protein (PLVAP), and potentially compensatory decrease in caveolin-1 (based on RNAseq data). Given that VEGF is a known permeability factor targeting junctions in the paracellular pathway [1, 25], we next tested if infection-induced HIF-1α/VEGF is required for bacterial transmigration and permeability at the BBB. Inhibition and knock-down of HIF-1α resulted in rescue of permeability and bacterial transmigration post-infection, thus demonstrating a critical role for HIF-1α/VEGF in bacterial transmigration and permeability at the BBB in meningitis.

RNAseq analysis also indicated regulation of several pathways related to HIF-1α and VEGF such as NF-kappaB, PDGFb and Ang/Tie2 signaling, as reported previously for RNAseq of brain microvessels from experimental stroke and glioma models [37, 16]. In the current meningitis data set, we observe an increase in angiopoietin 2 (Ang-2) and a decrease in Tie2 as well as protein tyrosine phosphatase receptor type B (PTPRB; one of the top down-regulated genes). We previously described that increased levels of Ang-2 contribute to BBB breakdown in healthy and ischemic mice which was rescued by activating Tie2 signaling via targeting PTPRB (also known as vascular endothelial protein tyrosine phosphatase VE-PTP) [27]. Interestingly, VE-PTP has been shown to be regulated by hypoxia in vitro in HUVECs and in vivo in a mouse model of oxygen-induced retinopathy [65], suggesting a similar regulation in meningitis as indicated by our sequencing data. Ang-1, the endogenous Ang-2 antagonist, has been reported to prevent VEGF-induced permeability by sequestering the tyrosine protein kinase Src via mDia. This leads to inhibition of VEGF downstream signaling via VEGF receptor 2 (VEGFR2) that requires activation of Src [26], whereas Ang-2 has been shown to be synergistic with VEGF in tumor angiogenesis and permeability including in brain tumors that are characterized by significant BBB damage [64]. A paracellular pathway involving β2-adrenoreceptor/β-arrestin via Src kinase leading to disorganization of junctional molecules has been reported for transmigration of *Neisseria meningitidis* across the BBB causing meningococcal meningitis [7]. Interestingly, β-arrestin-dependent endocytosis of VE-cadherin promoted by VEGF leads to paracellular permeability [25], which suggests that the upstream signaling in *N. meningitidis* transmigration might also involve HIF-1α/VEGF pathway in addition to β2-adrenoreceptor signaling. SNAI1 (Snail) has also been shown to contribute to BBB permeability in meningitis caused by GBS and potentially to bacterial transmigration [39] and this observation is supported by our RNAseq dataset showing twofold upregulation of Snail. Interestingly, Snail transcriptional activity via repression of VEGFR2 has been recently demonstrated in cancer resistance with beneficial outcome in tumor growth upon co-targeting of these pathways [47]. Potential interference of these pathways at the BBB in meningitis cannot be therefore ruled out.

Based on the in vitro data and RNAseq analysis that indicated a crucial role of HIF-1α pathway at the BBB in meningitis, we targeted the HIF-1α pathway for therapeutic rescue in a murine model of pneumococcal infection. Mice hematogenously infected with *S. pneumoniae* survived longer and with better clinical symptoms upon treatment with echinomycin, a potent HIF-1α inhibitor. While HIF-1α was indeed reduced in the treated animals, no difference was observed in the levels of *S. pneumoniae* either in the vessels or in the brain parenchyma. Analysis of blood–brain barrier function indicated reduced fibrinogen permeability and increased expression of tight junction proteins occludin and claudin-5, suggesting BBB improvement with echinomycin treatment. These results suggest that the primary mechanism of improved survival/clinal symptoms upon echinomycin treatment might be the result of improved BBB function upon inhibition of HIF-1α signaling. A bactericidal effect of echinomycin, however, can be ruled out as we observed similar levels of vascular bacteria in both echinomycin- and vehicle-treated groups. This is in line with a previous report employing echinomycin as a peptide antibiotic in a murine model of intraperitoneal *S. aureus* infection where the effective dose was 50–100 times of that applied in the current study [55]. Our in vitro bacterial growth data upon echinomycin treatment also support these results (not shown). At the low doses applied in the current study, inhibition of HIF-1α has however been shown to be effective in murine cancer models where echinomycin treatment resulted in increased survival [67, 73]. Similar to the in vitro findings, BBB tightening with echinomycin could lead to reduced bacterial numbers in the brain parenchyma at a particular time point post-infection compared to the untreated animals. The unchanged brain parenchymal bacterial load in our study can be explained by our analysis, which was at the survival end point as opposed to same time point for all animals. However, even with a minimal effect of echinomycin on early BBB tightening and bacterial transmigration and CNS deposition, the long-term effect of improved BBB function could potentially lead to reduced brain edema and alleviate the neurological damage.

As the neurological complications of seizures, stroke, long-term neurological disability and mortality are a result of initial BBB impairment and subsequent brain edema, therapeutic targeting of barrier function is a critical step in meningitis. Current strategies for bacterial meningitis utilize corticosteroids (mainly dexamethasone) as an adjuvant therapy in combination with antibiotics [3, 41]. In several cases, dexamethasone is administered alone even before antibiotics to control the BBB dysfunction that leads to increased cerebral edema and intracranial pressure. However, the benefit of dexamethasone in patient survival or neurological improvement was not evident in several clinical studies for various pathogens causing bacterial meningitis [4, 71, 22, 10, 53, 63]. In one study, dexamethasone therapy in HIV-associated *Cryptococcus neoformans* meningitis was associated with even more adverse events including disability than the control group [4]. New effective therapeutic strategies are therefore needed to target the BBB dysfunction associated with meningitis [3]. The benefit of dexamethasone has also been questionable in cancer therapy as elaborated in our recent review [20], where it is used in edema management particularly in brain tumors [19, 30, 66]. Interestingly, one mechanism proposed for the action of dexamethasone on edema reduction in glioma was via downregulation of VEGF leading to reduced permeability; the efficacy of dexamethasone, however, was dramatically reduced during hypoxia [50]. The insignificant effects of dexamethasone in meningitis can thus be explained by the above study combined with ours, demonstrating the role of hypoxia and HIF-1α/VEGF in meningitis. Regulation of angiogenesis and cancer-related pathways at the BBB in meningitis as indicated by bioinformatics analysis of our RNA sequencing data further support the role of HIF-1α/VEGF in meningitis similar to their role in cancer progression [14, 11, 16]. Several cancer therapeutics target HIF-1α/VEGF pathway alone or in combination with other pathways to alleviate tumor angiogenesis and permeability [21, 64, 16]. Given the limited effects of dexamethasone in meningitis and based on our study that demonstrates a critical role of HIF-1α/VEGF in meningitis at the BBB, we propose that targeting this pathway in meningitis as demonstrated using echinomycin in this study could lead to effective therapeutics for this deadly disease.

## Electronic supplementary material

Below is the link to the electronic supplementary material.

Supplementary file1 (PDF 27464 kb)

Supplementary file2 (MP4 868 kb)
