# Supplementary material for: HIF-1α is involved in blood–brain barrier dysfunction and paracellular migration of bacteria in pneumococcal meningitis
Source: Acta Neuropathol. 2020 Jun 11;140(2):183–208. doi: 10.1007/s00401-020-02174-2 (PMC7360668; doi:10.1007/s00401-020-02174-2)
Supplement: Supplementary file 1 — Supplementary file1 (PDF 27464 kb) [file 401_2020_2174_MOESM1_ESM.pdf]

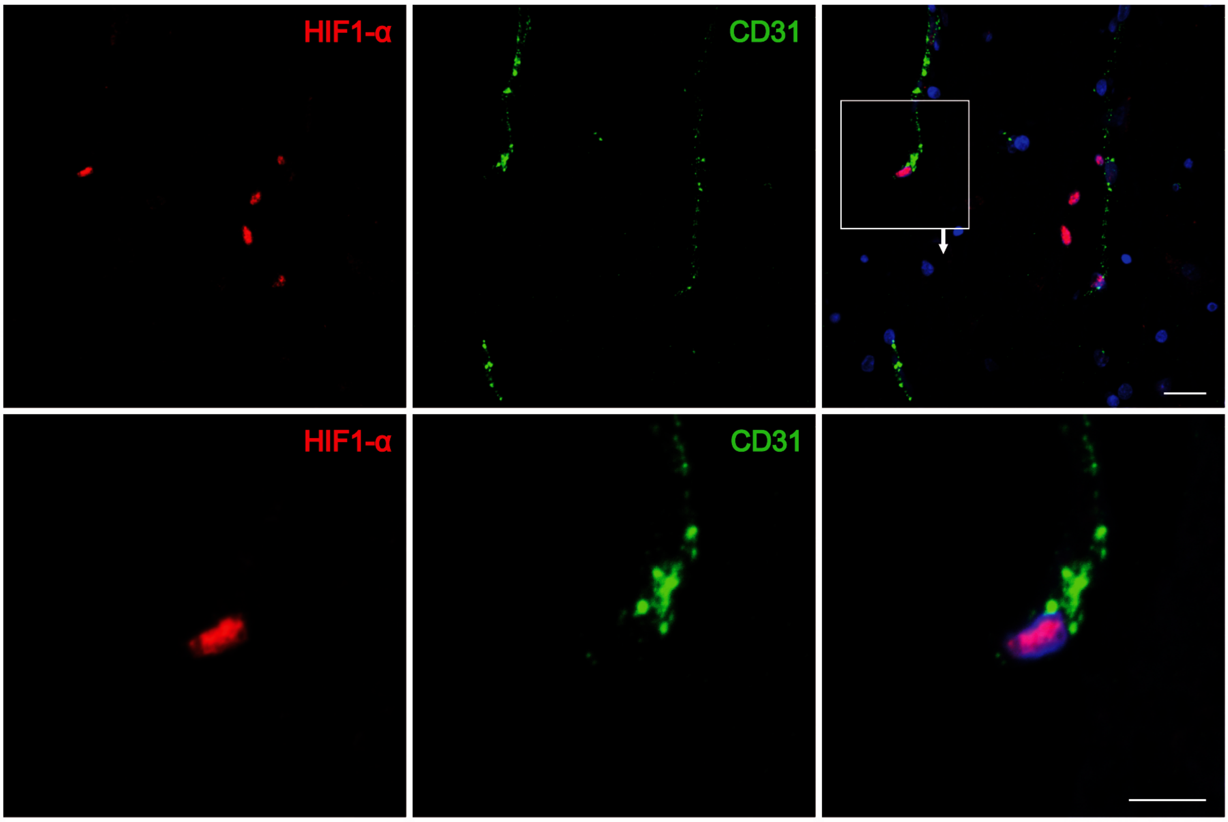

Figure S1. HIF-1α immunofluorescence staining of brain ECs in human pneumococcal meningitis.

Human brain paraffin specimen were subjected to HIF-1α immunofluorescence staining using CD31 as an endothelial marker. Top panel shows lower magnification (20 micron scale bar) of brain EC nuclei positive for HIF-1α whereas the bottom panel shows zoomed region of interest (scale bar 10 micron). Representative images were taken from 2 different cases out of total 4 cases that were positive for HIF-1α (table 1).

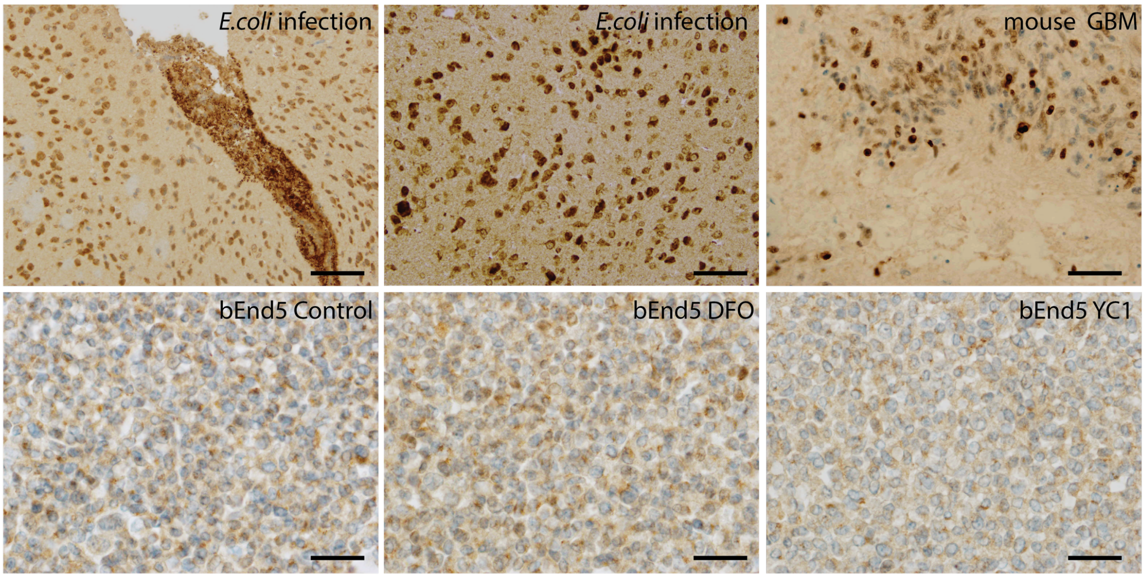

Figure S2. HIF-1α staining of other mouse brain meningitis cases including the staining controls.

Mouse brain paraffin specimen from *E. coli* meningitis (24 h *E. coli* K1, intracerebral) were also subjected to HIF-1α staining. Top left panel shows inflammatory infiltrate positively stained for HIF-1α, which was also observed in the cortex region (top middle). Representative images were taken from two different animals out of five animals analyzed. Mouse glioblastoma tissue (GBM) served as a positive control for HIF-1α staining as GBM is known to be associated with hypoxia and HIF-1α induction. The bottom panel shows cell pellets from bEnd5 cells that were formalin fixed and paraffin embedded and processed similar to other mouse brain tissues. While the controls do not show any nuclear HIF-1α, DFO treated cells clearly showed nuclear staining whereas treatment with YC-1 (HIF-1α inhibitor) showed the lowest signal. Scale bar: 50 µm - top panel, 25 µm - bottom panel.

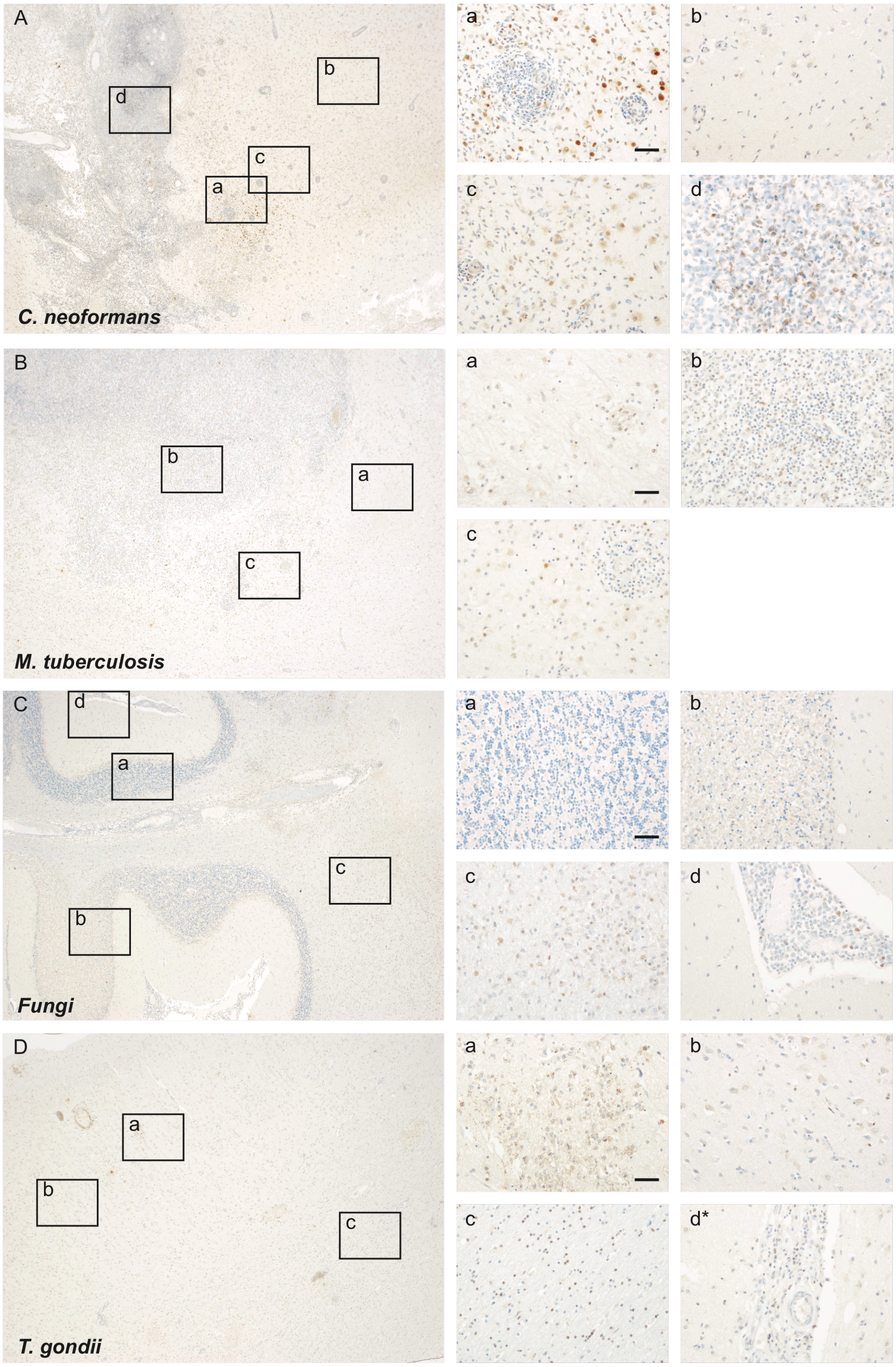

Figure S3. HIF-1α staining of other human meningitis cases.
Human brain paraffin specimen from different meningitis cases (indicated in the figure) were subjected to HIF-1α staining. Positive staining for HIF-1α was observed in different regions in all the specimens in vascular, neural and immune cells. The patient date of these cases is also included in **table 1**. Scale bar is 50 µm.

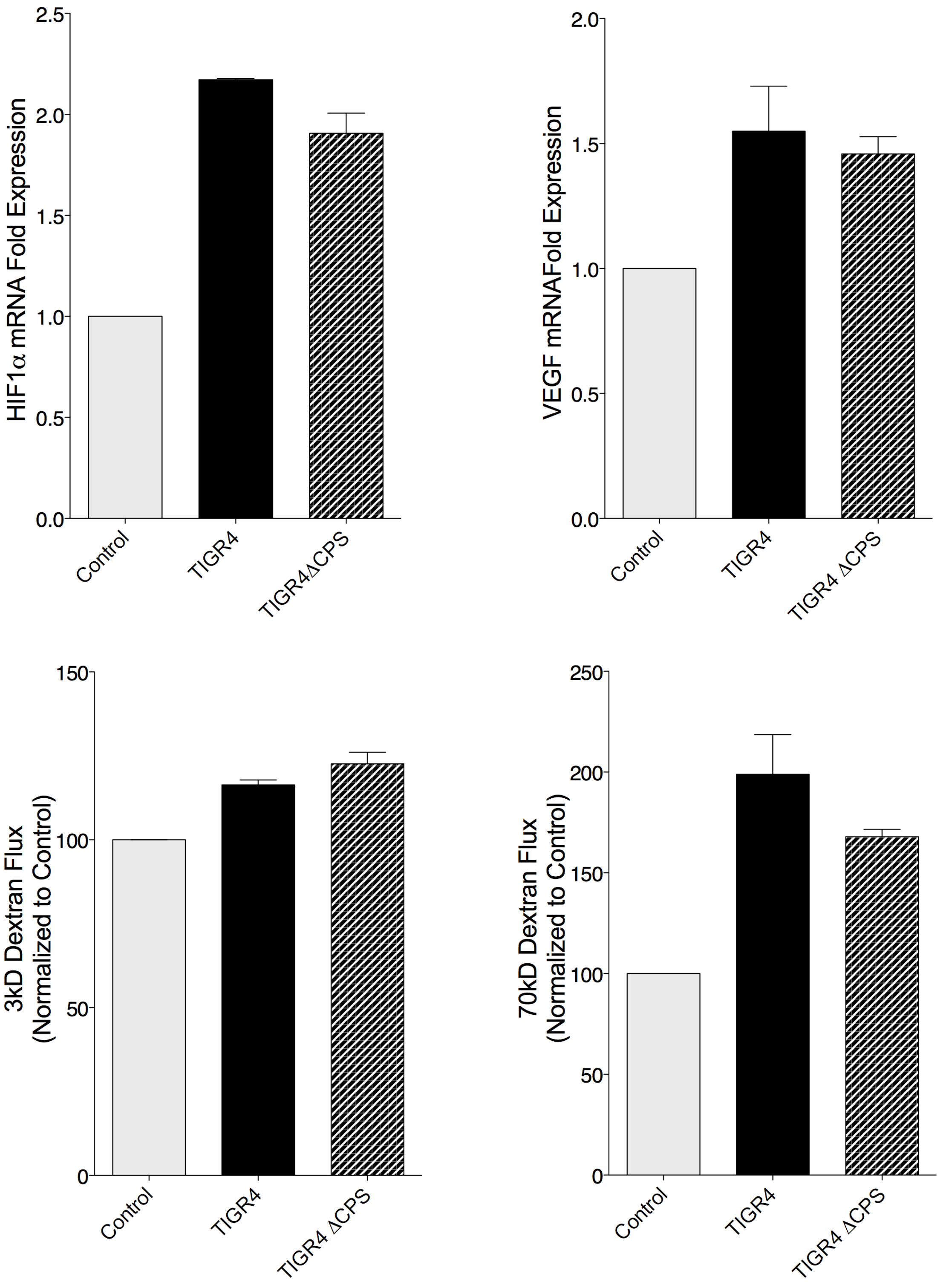

Figure S4. Role of *S. pneumoniae* capsule in HIF1α induction and permeability.
As the capsule of *S. pneumoniae* is critical for its virulence, we tested whether it is important for HIF1α induction and permeability. Using capsule deleted mutants of TIGR4 strain (Δcps), an induction of HIF-1α and VEGF similar to the wild-type strain was observed (top panel). Similar results were also obtained in dextran permeability experiments (bottom panel) suggesting no role of the *S. pneumoniae* capsule in HIF-1α induction and permeability. (Mean ± SEM, N=2 experiments using bEnd5 cells for both qRT-PCR/permeability assays.

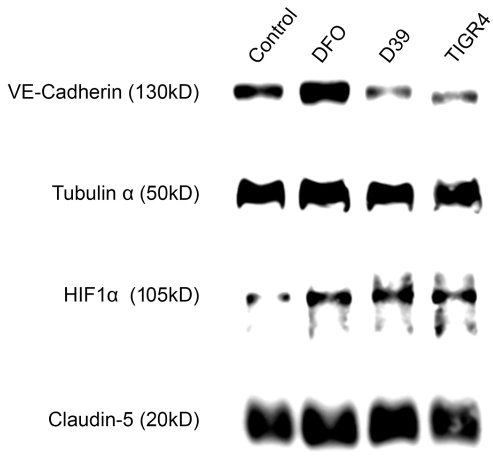

Figure S5. Loss of junction molecules upon *S. pneumoniae* infection in bEnd5 cells.
As *S. pneumoniae* infection of bEnd5 cells lead to permeability, Western blotting was performed to analyze junction molecules. VE-cadherin, an adherens junction marker was dramatically HIF-1α down regulated whereas clauin-5, a tight junction molecule was not altered. HIF-1α was upregulated as also shown in **figure 2**. Tubulin α, a house keeping protein served as the loading control. Representative Western blot from 2 experiments.

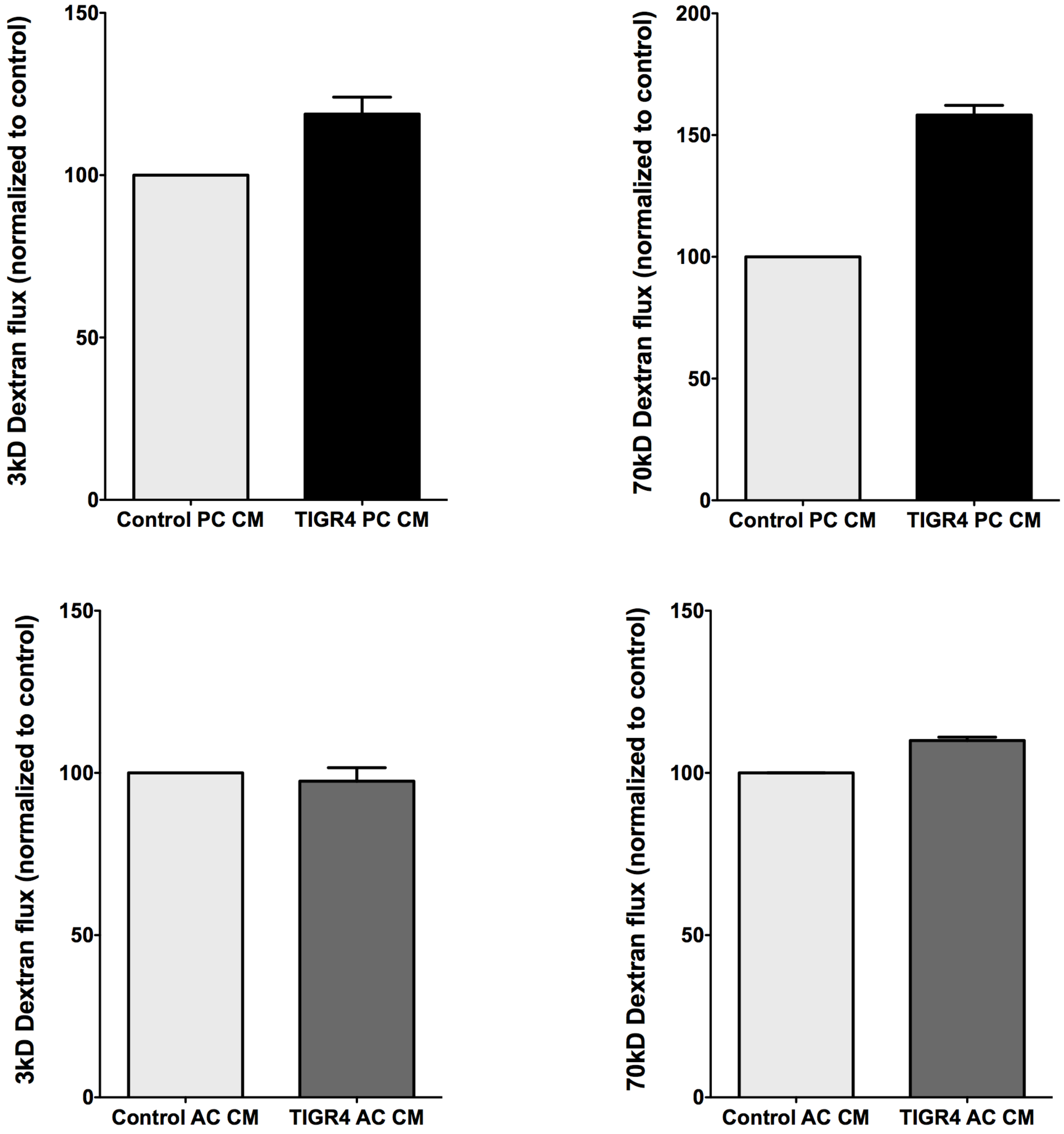

Figure S6. ***S. pneumoniae*** **infection of pericytes but not astrocytes causes EC permeability.** As *S. pneumoniae* infection of brain ECs lead to permeability, the influence of other NVU cells in permeability of EC barrier upon *S. pneumoniae* infection was tested *in vitro*. Conditioned media from primary human brain vascular pericytes (PC) at 3 h post infection with TIGR4 strain lead to increase in permeability of bEnd5 cells in tendency but not using the media from primary mouse astrocytes (AC) (Mean ± SEM, N=2 experiments).

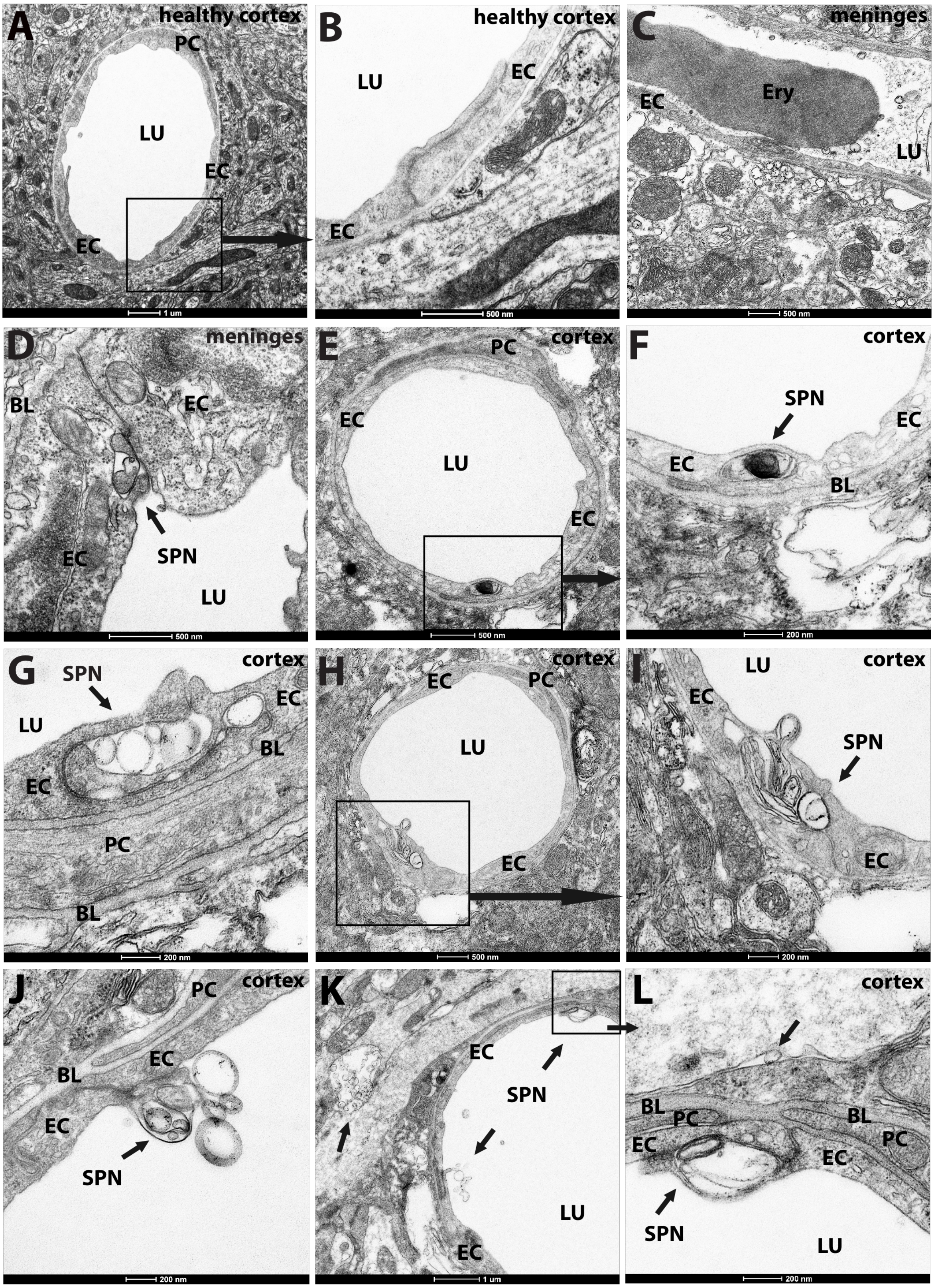

Figure S7. EM analysis of mouse brain vessels for localization of *S. pneumoniae*. Extended analysis of localization of *S. pneumoniae* (SPN) was performed by EM for several mice. In all mice, the localization of bacteria was primarily at the cell-cell junctions in cortex, and meninges (D-I) with visible extravasated bacteria (C, K, L). (N=6 animals analyzed using 3-5 thin sections from each animal). Figures C-I represent the uncolored version of figures 5 C-I. Arrows point to SPN at the junctions and in the parenchyma.

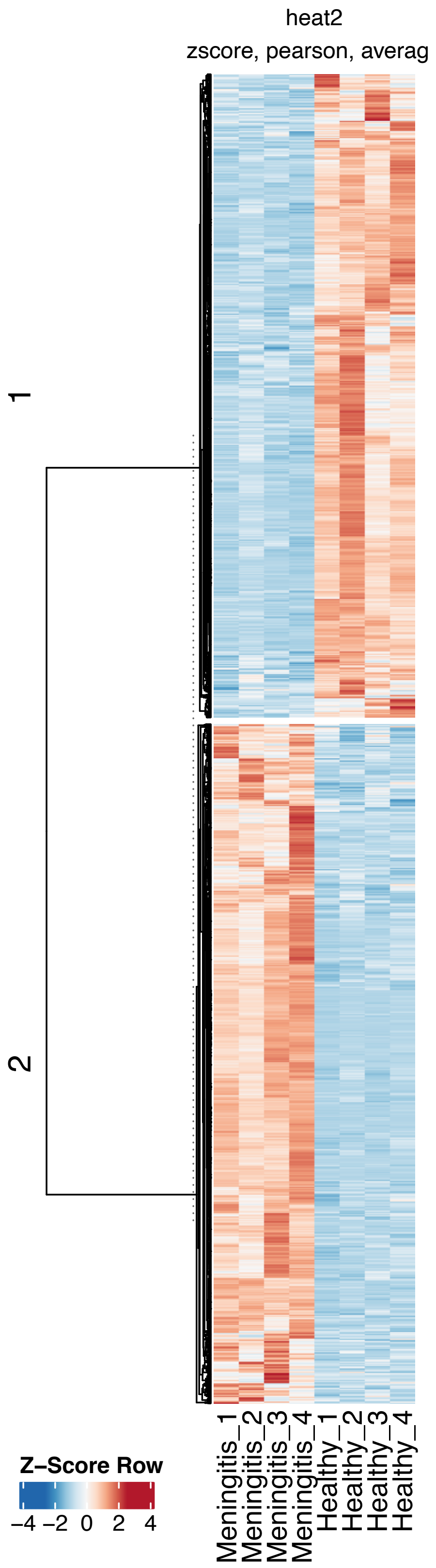

Figure S8. Heatmaps of hierarchical clusters of regulated genes in mouse brain vessels post pneumococcal meningitis. Hierarchical clustering by Euclidean distance similarity for all the up and downregulated genes is shown in the above heatmap by dendrograms. Cluster 1 indicates downregulated genes in meningitis whereas 2 indicates upregulated genes when compared to healthy controls.

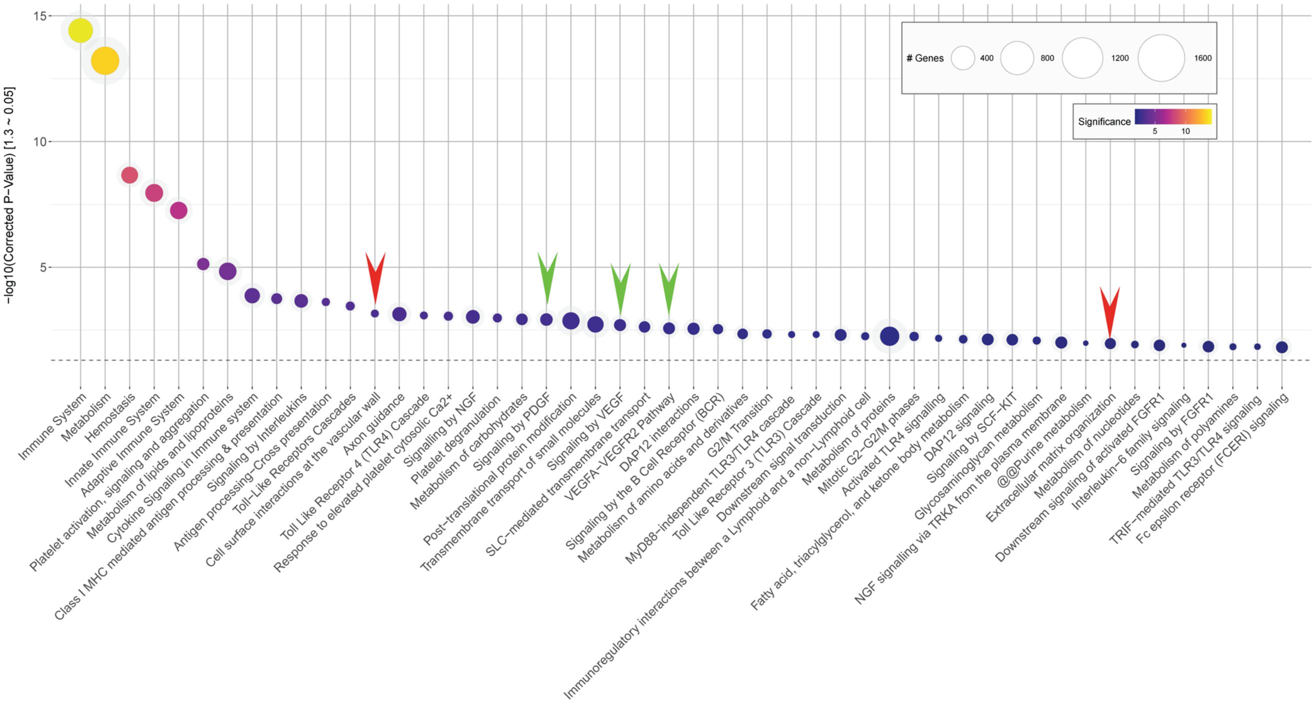

Figure S9. KOBAS reactome analysis of brain microvessels of *S. pneumoniae* infected mice.

S. pneumoniae infection of mice followed by isolation of microvessels and RNA sequencing was performed to study the mechanisms involved in transfer of *S. pneumoniae* across the BBB. Similar to KEGG and PANTHER analyses, KOBAS reactome analysis also indicated regulation of pathways related to cancer and inflammation. Pathways related to HIF-1α/VEGF signaling were also activated in the infected brain vessels when contrasted with healthy sham microvessels (green arrows) including regulation of cell adhesion molecules relevant to BBB permeability (red arrows). Genes significantly regulated per contrast (P-value < 0.05, Absolute (Log2FC) > 0.585) were included in the KOBAS enrichment.

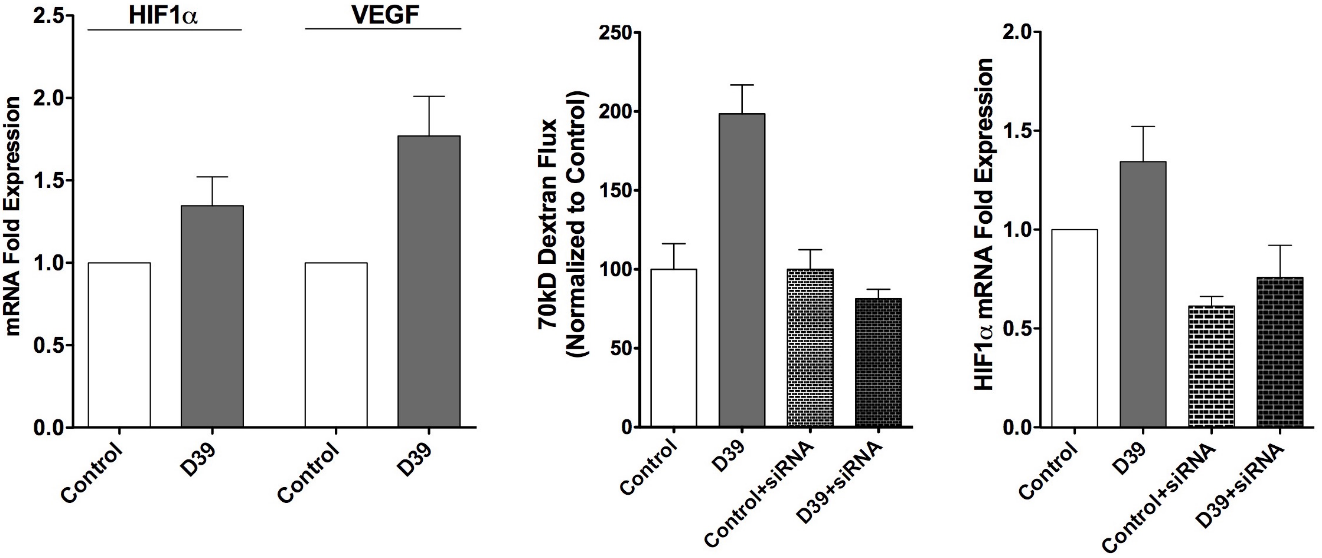

Figure S10. HIF-1α induction and permeability upon *S. pneumoniae* infection of human brain ECs.

S. pneumoniae infection of human primary brain ECs also lead to induction of HIF-1α and VEGF (left graph). Similar to mouse brain ECs, increase in permeability was observed in tendency for human brain ECs to the higher molecular weight 70 kD dextran. Moreover, the effect appeared to be HIF-1α dependent (middle graph) as it was abrogated upon *si*RNA knockdown of HIF-1α in these cells, which was confirmed by qRT-PCR analysis (right graph). (Mean ± SEM, N=2 for qPCR, and N=1 for permeability).

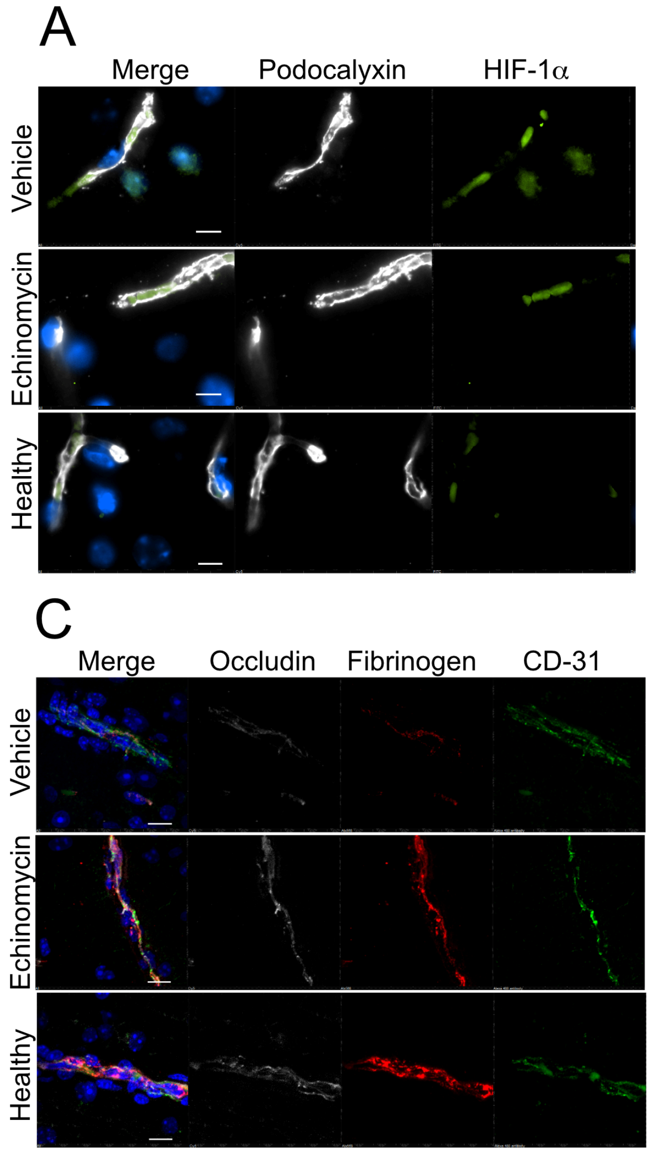

Figure S11. Echinomycin rescues BBB function over vehicle closer to healthy controls. *S. pneumoniae* infection of mice followed by echinomycin treatment leads to improved BBB phenotype compared to the vehicle treated mice. While **(A)** HIF-1α staining was decreased in echinomycin group, there was no change in **(B)** *S.pneumoniae* (Spn) staining. The expression of BBB functional markers **(C, D)** in echinomycin treated animals were closer to healthy controls indicating a therapeutic rescue when compared untreated vehicle group. The control healthy animals however were negative for Spn indicating the staining specificity (**B**, top right panel). Scale bar is 10 microns except top right panel where it is 2.5 microns. Control animal staining is representative of 6 animals.
